# Inferring pointwise diffusion properties of single trajectories with deep learning

**DOI:** 10.1016/j.bpj.2023.10.015

**Published:** 2023-10-17

**Authors:** Borja Requena, Sergi Masó-Orriols, Joan Bertran, Maciej Lewenstein, Carlo Manzo, Gorka Muñoz-Gil

**Affiliations:** 1 ICFO – Institut de Ciències Fotòniques, The Barcelona Institute of Science and Technology, Castelldefels (Barcelona), Spain; 2 Facultat de Ciències, Tecnologia I Enginyeries, Universitat de Vic – Universitat Central de Catalunya (UVic-UCC), Vic, Spain; 3 ICREA, Pg. Lluís Companys 23, Barcelona, Spain; 4 Institute for Theoretical Physics, University of Innsbruck, Innsbruck, Austria; 5 Institut de Recerca i Innovació en Ciències de la Vida i de la Salut a la Catalunya Central (IRIS-CC), Vic, Barcelona, Spain

## Abstract

To characterize the mechanisms governing the diffusion of particles in biological scenarios, it is essential to accurately determine their diffusive properties. To do so, we propose a machine-learning method to characterize diffusion processes with time-dependent properties at the experimental time resolution. Our approach operates at the single-trajectory level predicting the properties of interest, such as the diffusion coefficient or the anomalous diffusion exponent, at every time step of the trajectory. In this way, changes in the diffusive properties occurring along the trajectory emerge naturally in the prediction and thus allow the characterization without any prior knowledge or assumption about the system. We first benchmark the method on synthetic trajectories simulated under several conditions. We show that our approach can successfully characterize both abrupt and continuous changes in the diffusion coefficient or the anomalous diffusion exponent. Finally, we leverage the method to analyze experiments of single-molecule diffusion of two membrane proteins in living cells: the pathogen-recognition receptor DC-SIGN and the integrin α5β1. The analysis allows us to characterize physical parameters and diffusive states with unprecedented accuracy, shedding new light on the underlying mechanisms.

## Significance

Understanding the diffusion of particles in biological systems is crucial to unravel fundamental mechanisms in various fields of study. This research introduces a machine-learning method able to predict key physical properties, such as the diffusion coefficient and anomalous diffusion exponent, at each time step of the input trajectory. The method is especially well suited to characterize processes with either discrete or continuous diffusive changes without any prior information about the process. We validate it using synthetic trajectories and subsequently apply it to study the diffusion of membrane proteins in living cells. The findings provide unprecedented accuracy in the extraction of physical parameters and diffusive states, offering valuable insights into the underlying physical mechanisms of biological processes.

## Introduction

Advances in optical imaging have made it possible to observe single molecules in living biological systems (1). When combined with particle tracking algorithms, these techniques allow tracing the movement of individual molecules, viruses, and organelles with nanometric precision, enabling the study of transport mechanisms in complex biological environments. Through the biophysical characterization of trajectories, we can extract meaningful parameters to describe physical and biological processes, such as nanoscopic particles in the cell (2), active particles in complex environments (3), or even the motion of wild animals (4). However, accurately quantifying the trajectories remains a challenging task due to their stochastic nature and to experimental drawbacks, such as imaging noise and the emitter’s photophysics (5).

Nowadays, there exists a plethora of methods to characterize diffusive processes. The most common approaches are based on the calculation of the mean squared displacement (MSD; see Appendix A). Fitting the MSD offers an optimal way to estimate the diffusion coefficient from trajectories undergoing Brownian motion in many circumstances (6,7). However, studying anomalous diffusion trajectories is more challenging due to the intricate properties of the system’s dynamics, e.g., nonergodicity or aging (8), and artifacts associated to the presence of experimental noise (9). The anomalous diffusion exponent *α* can be faithfully extracted from the fit of the MSD averaged over an ensemble of trajectories (10). When dealing with ensembles of heterogeneous diffusers, individual information can be obtained by the time-averaged MSD (TA-MSD) for ergodic and sufficiently long trajectories (11). Additionally, when dealing with some specific diffusion models, we can exploit this knowledge to use methods based, for instance, on the power spectral density of single trajectories (12,13) or the Bayesian estimation of diffusive properties (14). Recently, as we extensively discuss below, machine-learning-based approaches (15) have been shown to boost these analyses.

**Figure 1:**
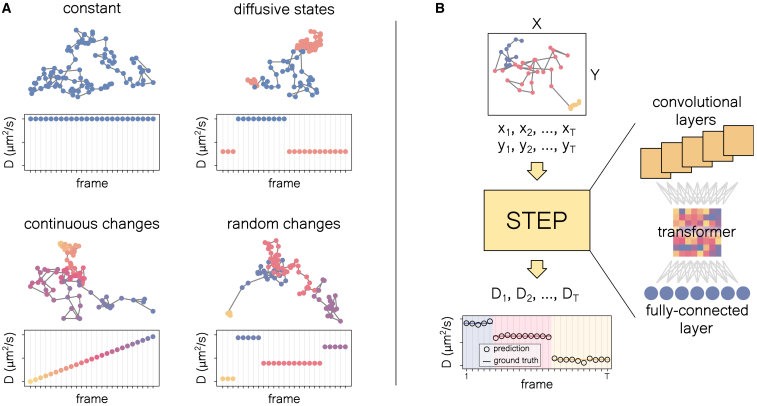
Heterogeneous trajectories and the STEP pipeline. (*A*) Examples of trajectories and the corresponding diffusion coefficient *D* as a function of time for constant *D*, changes within a discrete set of states with fixed *D*, continuous and monotonous change of *D*, and switch between random *D*s. (*B*) Schematic of the pipeline of STEP: an input trajectory is fed to the architecture, which consists of a stack of convolutional layers, a transformer encoder, and a pointwise feedforward layer. The model’s output is the pointwise prediction of the diffusion parameter of interest (in this case *D*). To see this figure in color, go online.

Nonetheless, the methods described above can only be used to study trajectories with constant diffusive properties. In cellular systems, a widespread diffusion feature is the occurrence of time-dependent changes of motion (16). Typically, these changes are associated with transient interactions with other components (17,18,19) resulting in the sudden variation of a parameter, e.g., the diffusion coefficient, which can switch between a discrete (20) and continuous set of levels (21,22,23). Furthermore, they can induce smooth changes such as those associated with the spatio-temporal heterogeneity of the environment (24). Examples of trajectories undergoing this kind of diffusion are schematically depicted in Fig. 1 *A*.

Trajectories with time-dependent diffusion properties pose an additional challenge to characterize the motion of individual particles, which has been tackled with different approaches. For trajectories displaying abrupt changes, the combination of statistical methods with segmentation algorithms (16,25,26) is a valuable strategy but cannot deal with long-range correlations and often offers limited time resolution due to temporal averaging. On the other hand, model-dependent methods such as the hidden Markov model have been quite successful in describing heterogeneous diffusion (27,28,29), although they require prior knowledge about the diffusive states involved and their kinetic scheme. Recently, data-driven approaches have shown remarkable capabilities to extract information from individual stochastic trajectories, even in the presence of changes of diffusion properties (15,30,31,32).

In this work, we propose STEP, a method based on state-of-the-art deep-learning architectures to extract pointwise diffusion features from individual trajectories without any prior information (see Fig. 1 *B*). This allows STEP to overcome many of the presented methods’ limitations, making it suitable for a wide range of applications. STEP features the most recent advances in sequence-to-sequence learning (33), which have shown impressive results in natural language processing tasks and beyond (34,35,36). It combines convolutional (37) and attention layers (38) to cope with the presence of short- and long-range correlations, providing remarkable performance over trajectories of any length and in the presence of noise.

The article is structured as follows: first, we introduce the main aspects of the proposed method. Then, we show the ability of STEP to predict diffusion properties, such as the diffusion coefficient and the anomalous diffusion exponent, on simulated data reproducing experimentally relevant scenarios. Then, we analyze simulated trajectories with smoothly varying diffusion coefficients, showing that STEP correctly finds the expected scaling. Finally, we use STEP to study two experimental datasets obtained by the tracking of single-molecule live-cell imaging experiments and reporting the motion of two membrane receptors: 1) the pathogen-recognition receptor DC-SIGN, which has been associated with random changes of diffusion coefficients (23), and 2) the α5β1 integrin, expected to be transiently arrested by binding to the cytoskeleton and the extracellular matrix (39). We conclude with a discussion of the results.

## Materials and methods

### The STEP architecture

Recently, we have witnessed an enormous effort in the development of deep-learning approaches to study diffusive processes (15). Previous works usually focused on characterizing diffusive properties of single trajectories (i.e., predicting an overall or average diffusive parameter for each input trajectory (40,41,42,43,44)). Recent works have proved the suitability of this approach to study complex phenomena in different experimental scenarios (45,46).

With STEP, we propose a sequence-to-sequence approach (33) that translates position coordinates into the diffusion properties of interest at every time step of the input trajectory, as illustrated in Fig. 1 *B*. In this way, the input and the output of the model have the same length. Although it is effectively impossible to characterize diffusion from a single displacement due to its stochastic nature, STEP uses the whole trajectory as context to perform the prediction at every point.

This approach allows us to study trajectories whose diffusion properties can vary over time with different patterns: from trajectories with constant diffusive properties to trajectories that sharply switch between different diffusive states, or with diffusive parameters that change continuously over time (see Fig. 1 *A* for examples). Unlike previous works, where expert input is needed to choose an appropriate method, STEP can be seamlessly applied to any diffusive data. Importantly, it does not rely on prior assumptions, such as the number of changepoints (47,48) or the properties of the expected diffusive states (49).

In diffusion phenomena, we deal with complex statistical signals that can exhibit various types of time correlations. Furthermore, we often encounter trajectories with very different lengths, even in the same experiment. Hence, it is crucial that the machine-learning models are length independent and able to capture correlations at different timescales to ensure that they are as applicable as possible. State-of-the-art architectures for diffusion characterization rely on very different approaches (50,51,52). Several models achieve outstanding performance by combining recurrent neural networks that account for long-range correlations (42,48) with convolutional neural networks that capture local features (46,53,54).

We propose an architecture combining convolutional and self-attention mechanisms. Interestingly, very recent works have shown analogous strategies, both with supervised (31) and unsupervised approaches (55). First, the input trajectory is processed by a series of convolutional layers that we build after the XResNet architecture (56). Then, the result follows through a transformer encoder (38), which can capture global correlations. Finally, we use a pointwise fully connected layer of nonlinear neurons to obtain the desired output dimension, as we illustrate in Fig. 1 *B*.

To produce the results presented in this work, we train two models: one to infer the diffusion coefficient *D* and another for the anomalous diffusion exponent *α*. Each is trained on simulated noisy trajectories that present abrupt changes in their respective diffusion properties (*D* or *α*). Throughout this work, we mainly consider two-dimensional (2D) trajectories, but the whole method can be easily adapted to any dimensions.

We provide a detailed description of the architecture, its training procedure, and the data in Appendix B. We also provide a library in Ref. (57) containing the code and extensive tutorials to reproduce the results of this work.

### Baselines

STEP provides pointwise diffusion properties for input trajectories, which, to the best of our knowledge, is a distinct task from that addressed by any existing method. In this context, it is challenging to perform a straightforward and equitable comparison of STEP with these methods, particularly considering that typical approaches involve the combination of various methods to achieve similar results.

**Figure 2:**
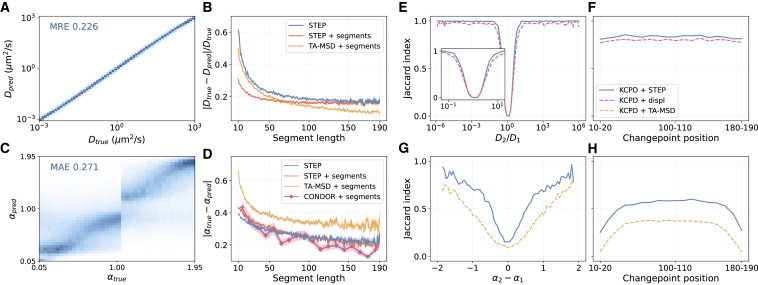
Time-dependent diffusion properties prediction. (*A*) 2D histogram of the predicted diffusion coefficient *D* compared to the ground truth. The mean relative error (MRE) over the whole test set is 0.226. (*B*) Relative error for the prediction of *D* as a function of the segment length. (*C*) 2D histogram of the predicted anomalous diffusion exponent *α* compared to the ground truth. The MAE over the whole test set is 0.271. (*D*) MAE for the prediction of *α* as function of the segment length. (*E*–*H*) Jaccard index for the changepoint detection problem as a function of (*E*) the ratio between consecutive segment *D*s, (*F*) the changepoint position for *D*, (*G*) the difference between consecutive segment *α*s, and (*H*) the changepoint position for *α*. For details about the data used in each panel, see Appendix B3 and Table I therein. To see this figure in color, go online.

Thus, instead of creating complex benchmarks, we compare STEP to the best alternatives proposed so far for the extraction of diffusion properties such as *D* and *α*. Since these methods cannot deal with time-dependent changes of diffusion, we apply them to trajectories pre-segmented according to the ground truth. For calculating *D* from Brownian trajectories, we employ the fitting of the TA-MSD, which is the optimal estimator for *D* in most cases (6). For estimating *α* from trajectories undergoing anomalous diffusion, we employ the TA-MSD fit in logarithmic space and CONDOR (47), recognized as the leading approach for this task in the AnDi Challenge (15). Although these methods benefit from a significant advantage due to the pre-segmentation, STEP typically achieves comparable performance (see Fig. 2).

## Results

### Pointwise prediction of diffusion properties

We first validate STEP on the task of inferring the pointwise diffusion coefficient from simulated trajectories reproducing transient Brownian motion with abrupt changes of diffusion coefficient. The diffusion coefficient can randomly vary in the range $D\in \left[10^{-3},10^{3}\right]$ and the dwell time in each diffusion coefficient is drawn from an exponential distribution between 10 and 190 (mean 57) time steps. Further details of the simulations and the datasets are described in Appendix B3. A 2D histogram of the ground truth versus the predicted diffusion coefficient shows that STEP can precisely determine the diffusion coefficient across its whole range (Fig. 2 *A*) with an overall relative error $\left|D_{\text{true}}-D_{\text{pred}}\right|/D_{\text{true}}=0.226$.

To further explore the performance of STEP, we calculate the relative error as a function of the segment length (i.e., the dwell time for each *D*; blue line in Fig. 2 *B*). The error increases for shorter segments, meaning that STEP needs sufficient statistics from surrounding points with similar properties to accurately predict the diffusive properties of a given point. Comparing these results with the TA-MSD’s prediction on pre-segmented trajectories, STEP is close to the optimal target performance (blue vs. yellow lines in Fig. 2 *B*). Notably, when STEP is provided with pre-segmented trajectories, we observe a further improvement, with a nearly twofold reduction of the error at short segment lengths (red line in Fig. 2 *B*), demonstrating outstanding prediction capabilities. However, it is outperformed by the TA-MSD baseline in long segments. We provide a deeper analysis about this performance trade-off in Appendix C, including a comparison to the Cramér-Rao lower bound (6).

We then examine the ability of STEP to predict the anomalous diffusion exponent *α*. We consider trajectories composed of segments following the same length distribution as in the Brownian motion case. Each segment is simulated using a different anomalous diffusion model and α∈[0.05,2] (see Appendix B3). The 2D histogram of the ground truth vs. the predicted *α* in Fig. 2 *C* shows that STEP successfully predicts the anomalous diffusion exponent. We obtain a mean absolute error (MAE) $\left|\alpha_{\text{true}}-\alpha_{\text{pred}}\right|=0.271$, which is in line with the top-scoring approaches for this task (48,53) in the anomalous diffusion challenge (15). As with most methods in the challenge, STEP is prone to errors for α∼1 (15). In addition, since several models are inherently only sub- or super-diffusive and the method tends to predict values of *α* within the training range, we observe a discontinuous behavior in the histogram for α∼1 (see Appendix A).

In Fig. 2 *D*, we report the MAE for *α* as a function of the segment length. STEP strongly outperforms the TA-MSD approach and shows a performance comparable to CONDOR. For this task, providing pre-segmented data to STEP marginally improves its performance for long segments, whereas it even reduces it for short ones. This result suggests that segment length is more important than the exact knowledge of the segment edges and STEP effectively combines local and global information.

In Appendix C and D, we extend the assessment of the performance of STEP as a function of the localization precision, the dwell-time duration, the number of segments in the trajectories, and the underlying diffusion model, showing that the method can be efficiently applied in a wide range of experimental conditions. Furthermore, we show that STEP correctly predicts α=1 for trajectories undergoing Brownian motion.

### Detecting diffusive changepoints in heterogeneous trajectories

For trajectories undergoing sudden changes of diffusion properties, the exact knowledge of the points at which these changes occur is crucial to infer temporal properties and kinetic rates of the system and fully characterizing the underlying physical process. Although STEP does not explicitly detect changepoints, its output provides a precise estimation of the diffusion property that is supposed to change, hence simplifying the task of changepoint detection and location with respect to the use of raw data. To highlight this capability, we compare the results obtained by a state-of-the-art kernel changepoint detection (KCPD) method (58,59) when applied to STEP’s predictions and to the time series of trajectory displacements. To assess the performance of the methods, we compute the Jaccard index (JI) considering as a true positive any changepoint predictions lying within a threshold distance E from the corresponding ground truth.

We first quantify the performance of the method to detect changes of the diffusion coefficient in heterogeneous Brownian motion trajectories. We use a benchmark dataset with trajectories of 200 time steps exhibiting a single changepoint and set E=5. Applying the KCPD algorithm on the prediction of STEP, we can successfully detect the changepoints with high accuracy. The detection improves as the differences between consecutive segments increase, achieving a nearly perfect detection for segments whose diffusion coefficients are just one order of magnitude apart, as we show in Fig. 2 *E* (blue line). Furthermore, the method is robust with respect to the changepoint position within the trace (Fig. 2 *F*, blue line). In contrast, when we apply KCPD directly over the trajectory displacements (dashed purple lines) we observe a decrease in performance over the whole range of diffusion coefficient ratios. On average, STEP produces a 20% reduction in error, reaching an average JI of 0.833 compared to 0.796 obtained with the raw displacements.

We perform a similar analysis to detect changes in the anomalous diffusion exponent. To ease the analysis, we consider only trajectories undergoing fractional Brownian motion (60). Since the anomalous diffusion exponent is an asymptotic property that cannot be easily calculated from the raw data, to build our baseline, we compute *α* with a linear fit of the TA-MSD on a log-log scale using a sliding window of 30 time steps, which we then feed into the KCPD algorithm (dashed yellow lines). Expectedly, the larger the differences between segment parameters, the better we can detect the changepoints, as we show in Fig. 2 *G*. We obtain a 30% error reduction by using STEP with respect to the baseline method, achieving an average JI of 0.515 and 0.297, respectively, with E=20. However, these metrics show that finding changes in *α* is significantly harder than in *D*. Moreover, we also observe a performance drop when the changepoints are near the trajectory edges (Fig. 2 *H*). In these cases, we deal with short segments whose anomalous diffusion exponent can be hard to determine, as they rely on the arising of long-range correlations.

### Revealing continuous changes of diffusion properties

When considering heterogeneous trajectories in the biological context, the typical behavior one expects is represented by particles undergoing diffusion with piecewise constant properties that can suddenly change, e.g., as the result of specific interactions with other biological components. However, the presence of molecular crowding and gradients of concentration can produce a continuous variation of diffusion properties over time. These changes might be challenging to detect due to the limited spatio-temporal resolution of the experiments or the lack of specific approaches for trajectory analysis. Since STEP predicts pointwise diffusion properties in a model-free fashion, it inherently features the capability to perform this kind of analysis, even without dedicated training.

To evaluate the performance of STEP on smoothly varying trajectories, we rely on simulations of scaled Brownian motion (SBM) (24). SBM trajectories are characterized by a time-dependent diffusion coefficient with a power-law relationship $D\left(t\right)∼t^{\alpha -1}$, where *α* is the anomalous diffusion exponent. Further details about simulations are given in Appendix B3.

**Figure 3:**
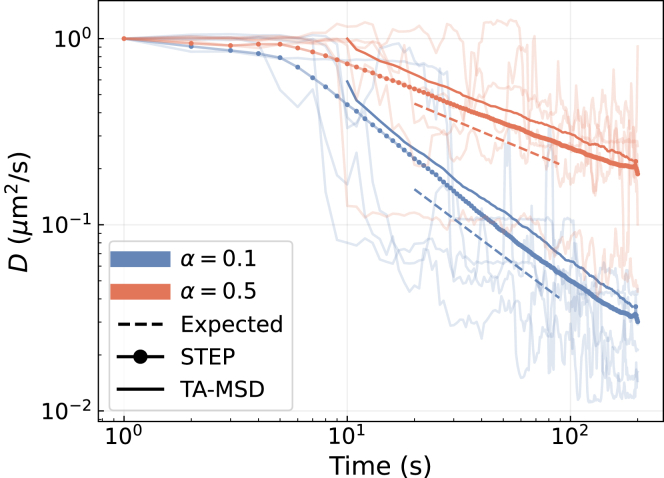
Continuous changes of diffusion properties. Predictions of the time-dependent diffusion coefficient of two sets of SBM trajectories with α=0.1 (blue) and 0.5 (red). The bold continuous lines show the average prediction over 3000 trajectories with STEP and a linear fit of the TA-MSD over a sliding window of 20 points. The thin continuous lines show a few example STEP predictions. The dashed lines indicate the theoretically expected scaling for every *α*. The lines have been normalized and shifted to compare their slopes easily. To see this figure in color, go online.

In Fig. 3, we show the predictions obtained for the diffusion coefficient at every time step of trajectories with α=0.1 and 0.5. The shaded lines represent the STEP predictions obtained for individual trajectories that, despite the fluctuations, already indicate the decreasing trend. Averaging over trajectories (round marker) reveals the correct power-law scaling (dashed lines). We also obtain the correct scaling with a linear fit of the TA-MSD on a sliding window of 20 points over the trajectories (solid lines). As shown, STEP can capture the scaling earlier, since it is not limited by the size of the window. Furthermore, the inference of *α* correctly provides a nearly constant value throughout the trajectory, as expected.

### Characterizing anomalous diffusion from changes of normal diffusive properties

**Figure 4:**
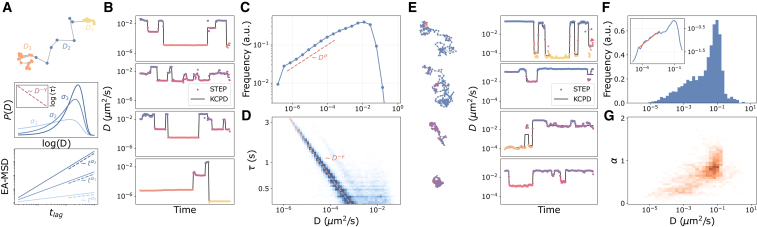
Switch between random diffusive states of the pathogen-recognition receptor DC-SIGN. (*A*) Characteristic features of the ATTM model: an exemplary trajectory undergoing changes of diffusion coefficient; a few examples of the distribution of *D* with different $\sigma_{i}$, and an example relation between the diffusion coefficient and the dwell time *τ* for a fixed *γ*; and the ensemble-average MSD scaling for the $\alpha_{i}=\sigma_{i}/\gamma$ that result from each of the previous $\sigma_{i}$ and a fixed *γ*. (*B*) Predictions of the diffusion coefficient obtained by applying STEP to simulated ATTM trajectories (dots) and the result of applying the changepoint analysis (black line). (*C*) Distribution of *D* obtained through the analysis described in (*B*), showing the expected power-law behavior at small *D*. (*D*) Relation between *D* and the dwell time *τ* obtained through the analysis described in (*B*), showing the expected power-law behavior. (*E*) Examples of experimental trajectories of DC-SIGN with the corresponding predictions obtained for *D* (dots) and the changepoint analysis (black line). (*F*) Histogram of the distribution of *D* obtained for the experimental trajectories. Inset: power-law fit at small *D*. (*G*) 2D histogram of *D* and *α* obtained for the experimental trajectories. For details about the data used in each panel, see Appendix B3 and Table I therein. To see this figure in color, go online.

To test the potential of STEP for the analysis of experimental trajectories, we use it to study the motion of the pathogen-recognition receptor DC-SIGN expressed in Chinese hamster ovarian cells (61). Previous analysis of these experiments revealed the occurrence of anomalous diffusion and weak ergodicity breaking as a consequence of stochastic changes of diffusion coefficient (23). This behavior was described in the framework of the annealed transit-time model (ATTM) (62), whose main features are schematically summarized in Fig. 4 *A*.

In brief, ATTM depicts Brownian diffusion randomly switching diffusion coefficient *D* for a given time *τ* (Fig. 4 *A*, top). This aims to mimic the spatio-temporal heterogeneities present in biological environments. Although particles are effectively performing Brownian diffusion at short scales, under particular distributions of *D* and *τ*, the diffusion is anomalous and weakly nonergodic at larger scales (62). For instance, one may consider that the values of *D* are sampled from a distribution with a power-law behavior $D^{\sigma -1}$ for small *D* and a fast decay for D→∞ (Fig. 4 *A*, middle). Moreover, assuming a correlation between *D* and the dwell time *τ* of the form $\tau \left(D\right)∼D^{-\gamma }$ (inset of Fig. 4 *A*, middle) to predict an anomalous diffusion exponent α=σ/γ (Fig. 4 *A*, bottom). Therefore, the correct characterization of both the distribution of *D* and *τ* is crucial to corroborate the compatibility with the underlying model. In the original work (23), changes of diffusivity were detected through a changepoint analysis (63) but the sensitivity and the time resolution of the method did not allow a thorough investigation of this behavior.

To demonstrate that STEP enables a better characterization of these data, we first use simulated ATTM trajectories. We set σ=0.3 and γ=0.4 (α=0.75), resulting in trajectories with $D\in \left(10^{-6.7},10^{0}\right)$ with 18 different segments, on average, for trajectories of 200 time steps. We segment the trajectories applying the KCPD algorithm introduced in the previous sections over the STEP predictions of *D*, as we show in Fig. 4 *B*. Thus, we assign to each segment a single *D*, taking the average segment prediction, and a *τ*. We successfully recover the power-law behavior of *D* (Fig. 4 *C*) and the power-law relationship between *τ* and *D* (Fig. 4 *D*). The faint harmonic in Fig. 4 *D* corresponds to $2D^{-\gamma }$, which results from the missed detection of a changepoint between consecutive segments with very similar *D* (hence similar *τ*). Interestingly, when performing predictions of *α*, STEP predicts α∼1, as expected from the properties of the diffusion model (see Appendix D).

Then, we apply this approach to the DC-SIGN trajectories of Ref. (23). The results confirm the occurrence of diffusivity changes between segments of nearly constant diffusion coefficient and with variable duration, as we show in Fig. 4 *E*. Interestingly, our approach reveals twice as many changepoints as the previous analysis.

The distribution of *D* obtained for trajectory segments spans several orders of magnitude, as we show in the histogram of Fig. 4 *F*. For small *D*, it displays a behavior compatible with a power law with exponent σ≈0.37 over nearly three decades (inset of Fig. 4 *F*), compatible with the ATTM. Notably, this behavior could not be directly verified in the original article. In principle, our method would allow us to verify the correlation between *D* and dwell time, as we have shown in the simulations. However, this task is limited by the variable trajectory length (64) and by the lack of statistics, in particular for segments at small *D*.

As a further test, we predict the anomalous diffusion exponent with STEP. We assign a single *α* by taking the average prediction of each segment. The results reported in Fig. 4 *G* show an interesting correlation between *D* and *α* that suggests a more complex diffusion pattern, involving the occurrence of anomalous diffusion also at the level of individual segments.

### Characterizing multi-state diffusion processes

We use STEP to analyze experimental trajectories of the integrin *α*5*β*1 diffusing in the membrane of HeLa cells (see Appendix E for experimental details). Integrins are transmembrane receptors for the extracellular matrix (ECM) in focal adhesions, which mechanically link the ECM and actin filaments in the cytoplasm and activate signaling pathways involved in cell migration, proliferation, or apoptosis (39). The dynamics of the integrin *α*5*β*1 is influenced by interactions with fibronectin and actin-binding proteins (64,65). Its motion has been reported to switch from fast free diffusion to slow free diffusion and immobilization, as well as exhibiting rearward actin-driven movement.

**Figure 5:**
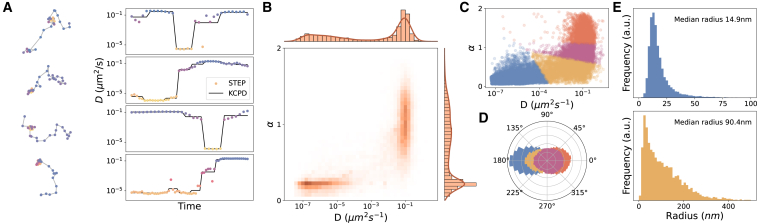
Multi-state diffusion of the integrin α5β1. (*A*) Examples of experimental trajectories of the integrin *α*5*β*1 with the corresponding predictions obtained for *D* (dots) and the changepoint analysis (black line). (*B*) 2D histogram of *D* and *α* with the respective marginal distributions. (*C*) Scatter plot of the predictions obtained for *D* and *α* at the segment level, color coded according to a clustering analysis performed with a *k*-means algorithm. (*D*) Distribution of the turning angle for the four clusters of segments obtained as in (*C*). (*E*) Distribution of the confinement radius for the clusters showing restrained diffusion. For details about the data in each panel, see Appendix B3 and Table I therein. To see this figure in color, go online.

We use STEP to predict both the diffusion coefficient and the anomalous diffusion exponent for the integrin *α*5*β*1 trajectories. Then, we segment the trajectories by applying the KCPD method to both predictions at once. In this way, we assign every segment a unique *D* and *α* by taking the average prediction over the segment. Examples of the results are shown in Fig. 5 *A* and the joint distribution of *D* and *α* in Fig. 5 *B*. The visual inspection of Fig. 5 *B* reveals two main clusters centered around ($D=10^{-6}\mu$m^2^/s, α=0.25) and (D=0.1μm^2^/s, α=1). The 2D histogram of the same parameters calculated at the pointwise level (pre-segmentation) does not show any major differences with respect to Fig. 5 *B*.

Nonetheless, combining the *k*-means clustering algorithm with the elbow method (66), we find the data optimally separates in four clusters of segments (Fig. 5 *C*) characterized by different motion features. The first two clusters show a rather restrained motion, with integrins spending 40% of the time in a state characterized by $D=1.2\times 10^{-5}\mu$m^2^/s and α=0.23, and 14% of the time with D=0.06μm^2^/s and α=0.46. For both clusters, the distribution of angles between successive steps shows a peak centered at 180°, indicating backward movements due to reflection at potential boundaries, as we show in Fig. 5 *D*. The confinement radius of the first cluster has a median of 14.9 nm (SD =13.3 nm), which is comparable to the localization precision of these experiments. This allows us to associate it with protein immobilization. The second cluster shows confined motion within areas with a broad distribution of sizes, as we see in Fig. 5 *E*, and a median radius of 90.4 nm (SD =98.2 nm). The third cluster represents 29% of the total recording and shows minor deviations from Brownian motion with α=0.88 and a nearly uniform angle distribution, and it has an average D=0.10μm^2^/s, close to the value typically reported for this protein. Interestingly, the analysis pinpoints a fourth population, corresponding to 20% of the total recording, undergoing superdiffusion with α=1.3 and D=0.14μm^2^/s, and with a persistent direction of motion between consecutive steps (Fig. 5 *D*).

## Discussion

In this work, we present STEP, a machine-learning method to predict diffusion properties from individual trajectories at every time step. The method relies on a combination of state-of-the-art machine-learning architectures that take into account correlations at different timescales. The presented approach is especially appealing to analyze trajectories from particles undergoing heterogeneous motion, where changes in diffusion properties occur over time. Moreover, it does not require prior knowledge of the underlying physical process or the temporal resolution at which changes in diffusion occur.

To illustrate the power of STEP, we benchmark it on simulated trajectories under various conditions. We show its ability to predict piecewise constant diffusion properties, such as the diffusion coefficient or the anomalous diffusion exponent, in noisy and short trajectories. Furthermore, we demonstrate that STEP boosts the accuracy of a changepoint detection algorithm to detect the time at which diffusion changes take place. Importantly, we also prove the suitability of our method to study continuous changes of diffusion.

To further showcase the potential applications of the method, we study trajectories obtained by tracking live-cell single-molecule imaging experiments of proteins of the plasma membrane. First, we characterize the motion of the pathogen-recognition receptor DC-SIGN, which was shown to exhibit random changes in the diffusion coefficient. Our analysis confirms such a hypothesis and improves the accuracy with which we detect these changes. Moreover, our results suggest the occurrence of more complex phenomena that need further investigation. Second, we study the diffusion of the integrin $\alpha_{5}\beta_{1}$. In agreement with previous works, our analysis confirms the existence of different diffusion modes and allows their precise classification according to the diffusion coefficient, the anomalous diffusion exponent, and the levels of spatial constraint.

We believe that STEP represents a first step toward a new class of machine-learning algorithms to study dynamic systems through a sequence-to-sequence approach. The instantaneous prediction of the property of interest enables the characterization of the trajectories at experimental time resolution without averaging and filtering and minimizes the prior knowledge needed to perform the analysis. As such, the results obtained with STEP can provide information about diffusion properties with unprecedented resolution and thus shed light on the underlying physical processes of a variety of systems. One of the primary advantages of STEP is its broad applicability. However, as demonstrated in the “results” section, specialized methods may produce more accurate results when applied specifically to their intended tasks. Consequently, a significant benefit of STEP is its potential integration with these methods, facilitating their utilization across a wider spectrum of scenarios, such as enhancing trajectory segmentation.

## Data and code availability

All the resources relative to the machine-learning model are accessible in the public repository from Ref. (57). The experimental data are available upon request.

## Author contributions

Conceptualization, B.R. and G.M.G.; methodology, B.R., G.M.G., and C.M.; software, B.R.; investigation, S.M., J.B., and C.M.; formal analysis, B.R., G.M.G., and C.M.; supervision, G.M.G., M.L., J.B., and C.M.; writing, B.R., G.M.G., M.L., and C.M.

## Supporting material

Document S1. Figures S1–S4, Table I, and Appendix A–E

Document S2. Article plus supporting material
