# Supplementary material for: Inferring pointwise diffusion properties of single trajectories with deep learning
Source: Biophys J. 2023 Oct 17;122(22):4360–9. doi: 10.1016/j.bpj.2023.10.015 (PMC10698275; doi:10.1016/j.bpj.2023.10.015)
Supplement: Document S1. Figures S1–S4, Table I, and Appendix A–E [file mmc1.pdf]

Biophysical Journal, Volume *122*

Supplemental information

Inferring pointwise diffusion properties of single trajectories with deep learning

Borja Requena, Sergi Masó-Orriols, Joan Bertran, Maciej Lewenstein, Carlo Manzo, and Gorka Muñoz-Gil

Appendices of "*Inferring pointwise diffusion properties of single trajectories with deep learning*"

Borja Requena,[1] Sergi Masó,[2] Joan Bertran,[2] Maciej Lewenstein,[1,3] Carlo Manzo,[2,*] and Gorka Muñoz-Gil[4,†]

[1]*ICFO – Institut de Ciències Fotòniques, The Barcelona Institute of Science and Technology, Av. Carl Friedrich Gauss 3, 08860 Castelldefels (Barcelona), Spain*

[2]*Facultat de Ciències, Tecnologia i Enginyeries, Universitat de Vic – Universitat Central de Catalunya (UVic-UCC), C. de la Laura,13, 08500 Vic, Spain*

[3]*ICREA, Pg. Lluís Companys 23, 08010 Barcelona, Spain*

[4]*Institute for Theoretical Physics, University of Innsbruck, Technikerstr. 21a, A-6020 Innsbruck, Austria*

Appendix A: Diffusion properties

In this section, we briefly highlight some of the main characteristics of normal and anomalous diffusion. We refer the reader to Refs. [1, 2] for a nice and thorough introduction to the field.

Diffusion trajectories are often described by means of their mean squared displacement (MSD) which, in the case of Brownian motion, shows a linear scaling with time, i.e. MSD $\propto Dt$, where D is the diffusion coefficient. However, there can be deviations from such linear scaling, resulting in a power-law relation between the MSD and time, i.e. MSD $\propto K_\alpha t^\alpha$, where α is defined as the anomalous diffusion exponent and K_α is an effective diffusion coefficient. The former allows us to distinguish between normal (or Brownian) diffusion ($\alpha = 1$) and anomalous diffusion ($\alpha \neq 1$).

The appearance of anomalous diffusion can be associated with very different phenomena, from the arising of correlations in the motion of the diffusing particle to the presence of spatiotemporal heterogeneity. To account for most of these phenomena, we follow Ref. [3] and consider five anomalous diffusion models with specific ranges for the anomalous diffusion exponent: annealed transient time model (ATTM) [4] with $\alpha \in [0.05, 1]$, continuous-time random walk (CTRW) [5] with $\alpha \in [0.05, 1]$, fractional Brownian motion (FBM) [6] with $\alpha \in [0.05, 1.95]$, Lévy walk (LW) [7] with $\alpha \in [1.05, 2]$, and scaled Brownian motion (SBM) [8] with $\alpha \in [0.05, 2]$.

Appendix B: Machine learning pipeline

Here, we provide a detailed explanation of the machine learning approach followed to obtain the results described throughout this work.

As we briefly mention in the main text, we train two different models: one for the diffusion coefficient task, and one for the anomalous diffusion exponent. We report the results regarding the prediction of the diffusion coefficient and anomalous diffusion exponent in Results. We implement both models following the same principles with very minor differences. In this section, we describe the architecture and the training process that we follow and, when needed, highlight the differences between models.

We provide the source code with extended explanations on how to reproduce the results in [9]. We make extensive use of the *PyTorch* [10] and *fastai* [11] libraries to implement the architecture and the training procedure. The kernel changepoint detection method (KCPD) was implemented using the *ruptures* Python library [12].

1. Architecture details

We propose to use a model that takes a trajectory $\mathbf{x}$ as input and outputs the target diffusion properties at each time step. The input trajectory is a d-dimensional vector of arbitrary length T, whose elements, x_t, correspond to the particle position at every time step t. Then, the output is a one-dimensional vector of length T, whose elements correspond to the diffusion property of interest at every time step, e.g., D_t in the case of the diffusion coefficient. See Fig. 1 for further details about the dimensions. Throughout this work, we mainly consider trajectories of dimension $d = 2$.

The model we propose consists of three main modules: an initial convolutional part that processes the input trajectory; a self-attention-based part that feeds on the features extracted by the previous one; a shallow pointwise fully connected feedforward module that provides the desired output dimensions. The entire architecture is length independent, which allows us to process trajectories of arbitrary lengths.

Convolutional module – The first main convolutional module allows us to expand the trajectory dimension with several convolutional filters. This provides the following layers with a richer embedding based on short-range correlations.

We build it following the XResNet [13] architecture. As we show in Fig. 1, it consists of an initial convolutional layer, commonly referred to as the *stem*, followed by a series of *residual blocks* that feature a convolutional layer with a skip connection. We use one-dimensional convolutions with a kernel size of three and stride one to preserve the trajectory size. However, we use a

* carlo.manzo@uvic.cat
† munoz.gil.gorka@gmail.com

kernel size of one in the skip connections, which can act as the identity or a scaling factor whose main purpose is to match the tensor shapes on both paths of the residual blocks, as we explain below. This module can take a batch of input trajectories of size [`batch_size` $\times T \times d$] and output a batch of features of size [`batch_size` $\times T \times$ `embedding_size`].

Throughout the architecture, we add a batch normalization layer directly after every convolutional layer, and we use the rectified linear unit (ReLU) activation function by default, except in the last output layer.

To produce the results, we use a single convolutional layer and a ReLU activation in the stem. We use 64 filters to predict the diffusion coefficient and 32 filters for the anomalous diffusion exponent. Then, we have added three residual blocks with 128, 256 and 512 filters, respectively. Hence, `embedding_size` = 512. In these blocks, the convolutional paths have two convolutional layers: the first one increases the embedding size and the second one preserves the dimensions. In the skip connection, we only have one convolutional layer that increases the embedding size to match the dimensions of the convolutional path. We implement the ReLU activation at the end of the block, after we add the outcome of both paths.

Self-attention module – We process the features extracted by the convolutional module with a self-attention mechanism that allows the model to capture long-range correlations.

More precisely, we implement a *transformer encoder*, as it was introduced in Ref. [14]. As we illustrate in Fig. 1, the encoder block has two main parts, both featuring a skip connection followed by a layer normalization after the sum of both paths. In the first one, we have a multi-head attention layer that feeds on the input and, in the second one, we have a couple of pointwise feedforward layers, which are equally applied to each element in the incoming tensor. Furthermore, we can add a positional encoding before the first encoder block, which provides information about the relative position of each element in the trajectory. This module can process a batch of embeddings preserving its dimensions. Hence, the input and the output both have size [`batch_size` $\times T \times$ `embedding_size`].

To produce the results, we use four transformer encoder blocks with eight heads in the multi-head attention layers. The pointwise feedforward part adds two fully-connected layers with `embedding_size` neurons each, i.e, 512 in this case. Interestingly, we have found that, after the convolutions, the positional encoding has very little impact on the results. Therefore, in the interest of simplicity, we have not used it to obtain the results reported in this work.

Feedforward module – The last main part is a shallow feedforward fully-connected network that acts element-wise on the features extracted by the previous module. We tailor this part to the specific task at hand

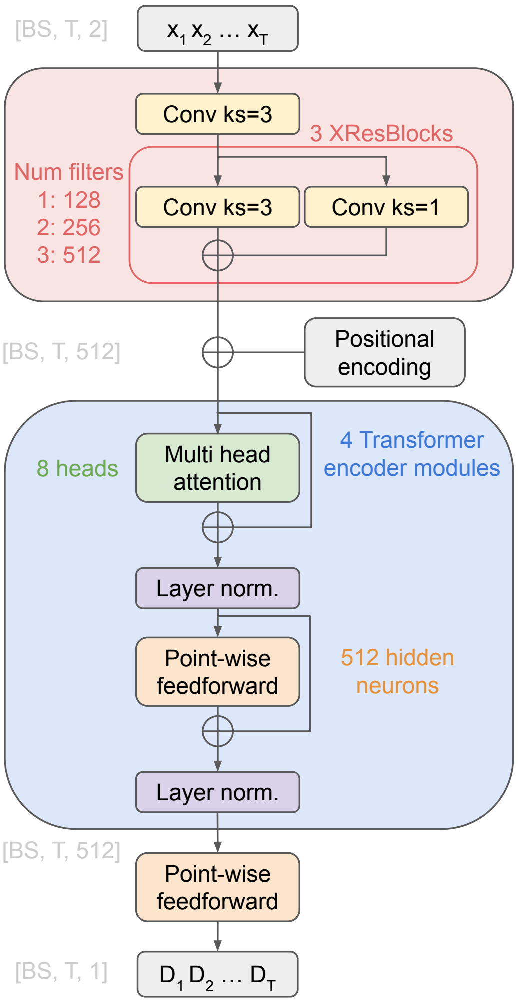

Figure 1. Machine learning architecture representation

to achieve the desired output with the proper dimensions.

For instance, in a regression task, the output dimension is one and we use a scaled sigmoid activation function at the end to define the output range with some margin, e.g., $\log D \in (-3.1, 3.1)$, $\alpha \in (0, 2.05)$. This margin allows the sigmoid to reach the desired values before it saturates. In a hypothetical case of classification task (as e.g. classifying between diffusion models as done in Ref. [3]), the final dimension is the number of classes and we use a softmax activation function. Then, we obtain the predictions by choosing the class with the maximum activation value. Hence, we can process a feature batch of size [`batch_size` $\times T \times$ `embedding_size`] and output their predictions with size [`batch_size` $\times T \times$ `num_class`]. In case that `num_class` > 1, as in a classification task, we perform an additional post-processing step to obtain an output of size [`batch_size` $\times T \times 1$] with the corresponding predictions at each time step.

2. Training procedure

We follow a standard gradient-based training procedure for both of our models. The only differences between them arise from the training data and how we process it.

The main training loop consists on:

1. Predict the values over a batch of training data.
2. Compute the loss function with respect to the true values.
3. Update the model parameters based on the loss gradient.

We use batches containing 128 trajectories and the L1 loss function, which corresponds to the mean absolute error. Formally,

$$\mathcal{L}_{\mathrm{MAE}}(\mathbf{x}) = \frac{1}{n\sum_i T_i}\sum_{i=1}^{n}\sum_{t=1}^{T_i}|y_{i,t} - f(\mathbf{x}_i)_t| \ , \tag{B1}$$

where $f(\mathbf{x}_i)_t$ denotes the prediction of the i-th trajectory at the time step t in a batch of n trajectories. $y_{i,t}$ denotes its true label for the same time, and T_i denotes its length.

To perform the parameter update, we use an Adam [15] optimizer. We use the fastai [11] library to choose the learning rate with the learning rate finder tool, typically of the order of 10^{-4}. Then, we implement a schedule over the training batches both in the learning rate and its momentum, following the one-cycle policy introduced in Refs. [16, 17]. We train our models until the performance in the validation set stabilizes, typically between ten to twenty epochs.

To further prevent overfitting and enhance the model generalization capabilities, we use dropout [18] and weight decay [19, 20]. Additionally, we add Gaussian localization noise at different intensities to the trajectories as a form of data augmentation.

3. Data

In order to properly evaluate our models, we generate several independent data sets. We use one to train and validate our models, and we use the others to test them on unseen scenarios. All the results that we report throughout this work are obtained using the test sets, which we design to evaluate different aspects of our models.

In Table I, we provide the details about the data sets that we use to train, validate and test our models. These data sets contain simulated trajectories with their corresponding labels at each time step. We have two main approaches to simulate the trajectories depending on whether we deal with normal or anomalous diffusion. Below, we explain how we generate the data for both cases.

While there are some differences between how we simulate and label our trajectories for normal and anomalous diffusion, there are several common factors that hold for all of them. For instance, all segments have constant diffusion properties and they are, at least, 10-time steps long.

Brownian motion – We simulate Brownian motion trajectories by taking uncorrelated Gaussian noise as the trajectory displacements. We control the diffusion coefficient at each time step with the standard deviation of the Gaussian noise, which corresponds to $\sqrt{2D}$. This way, we can easily generate segments of arbitrary lengths with a constant diffusion coefficient, D, along the trajectories. Finally, we perform the cumulative sum of the displacements to obtain the trajectory coordinates and we subtract the initial position such that they start at the origin.

We consider diffusion coefficients across six orders of magnitude $D \in [10^{-3}, 10^{3}]$. However, we take its logarithm as labels for the regression task, such that $y_i \in [-3, 3]$ at every time step. This greatly simplifies the problem and allows us to keep a consistent performance across all orders of magnitude.

Additionally, we can simulate experimental localization noise by adding Gaussian noise with standard deviation σ_{noise}. We use this as a form of data augmentation during training and to study the model's resilience to noise. See Table I for further details.

Anomalous diffusion – To simulate anomalous diffusion trajectories, we consider the five diffusion models introduced in Appendix A with their respective anomalous diffusion exponent ranges. We generate full trajectories for each model following the same procedure detailed in the Supplementary Material from Ref. [3] and using the library provided by the authors [22]. Then, in order to obtain heterogeneous trajectories, we split them into segments and combine them together. We impose the condition that two consecutive segments must differ, at least, either in the diffusion model or the anomalous diffusion exponent. Finally, we add Gaussian localization noise, with standard deviation σ_{noise}. Then, we normalize the resulting displacements by their standard deviation and subtract the initial position to ensure that the trajectory starts at the origin.

Therefore, we have two labels at each time step: the anomalous diffusion exponent and the diffusion model with which the corresponding segment was generated. This allows us to use the same data for both a regression task in the anomalous diffusion exponent and a classification task in the diffusion model. However, in this work, we have mainly focused on the first one. Furthermore, we balance all the data sets such that there is an even representation of both the anomalous diffusion exponents and diffusion models throughout all the time steps.

Task	Models	D	α	σ_{noise}	Traj. length	Segments	Seg. length	Size
**Train BM**	Brownian motion	$[-3, 3]$	1	$[-6, 2]$	200	$[2, 5]$	$[10, 190]$	100,000
**Train AnDi**	all anomalous	1	$[0.05, 2]$	0.1	200	$[2, 5]$	$[10, 190]$	100,064
Fig. 2A & B, Fig. 3D	Brownian motion	$[-3, 3]$	1	0	200	$[2, 5]$	$[10, 190]$	48,000
Fig. 2C, Fig. 4 Fig. 3C & D	all anomalous	1	$[0.05, 2]$	$[-5, 2]$	200	$[2, 5]$	$[10, 190]$	49,994
Fig. 2D, Fig. 3A & B	all anomalous	1	$[0.05, 2]$	$\{0, 0.1\}$	200	$[2, 5]$	$[10, 190]$	50,000
Fig. 2E & F	Brownian motion	$[-3, 3]$	1	0	200	2	$[10, 190]$	50,000
Fig. 2G & H	FBM	1	$[0.05, 1.95]$	0	200	2	$[10, 190]$	40,000
Fig. 3	SBM	1	$\{0.1, 0.5\}$	0	200	1	200	6,000
Fig. 4B, C & D	ATTM	(-6.7, 0)	0.75	0	200	$[1, 51]$	$[1, 200]$	10,000
Fig. 4E, F & G	(experiment)				$[200, 2000]$			755
Fig. 5	(experiment)				$[20, 500]$			4734
Fig. 2A	Brownian motion	$[-3, 3]$	1	0	$[20, 660]$	$[1, 11]$	$\{20, 40, 60\}$	22,000
Fig. 2B	Brownian motion	$[-3, 3]$	1	0	$[40, 660]$	$[2, 11]$	$\{20, 40, 60\}$	20,000
Fig. 2C	Brownian motion	$[-3, 3]$	1	$[-6, 0]$	200	$[2, 5]$	$[10, 190]$	48,000
Fig. 2D	Brownian motion	$[-3, 3]$	1	0	200	[2, 5]	[10, 190]	200,384

Table I. **Data set details for all the results reported throughout this paper.** The ranges for D and σ_{noise} are in $\log_{10}$ scale, and we take α intervals of 0.05 within the denoted ranges. The values of D are logarithmically spaced and we take 1000 unique values unless stated otherwise. All the datasets with 2 to 5 segments have their lengths sampled according to an exponential distribution with a minimum length of 10 and a maximum of 190 steps, with an average of ~ 57 time steps. We use 20% of the training data (first two rows) for validation and hyperparameter tuning, whereas the rest is used for testing. We use the same Brownian motion test set from Fig. 2A & B to predict α for Fig. 3D. We take two independent sub-samples of a test set with 199,976 trajectories: one for Fig. 2C, Fig. 3C & D, Fig. 4 and the other for Fig. 2D, Fig. 3A & B. In Fig. 2D, we consider noiseless trajectories, although we add noise with $\sigma_{\text{noise}} = 0.1$ to flat CTRW segments that would result in numerical instabilities for the TA-MSD method. To generate the noisy trajectories for Fig. 2C, Fig. 3C & D and Fig. 4, we add 128 random levels of localization noise to each trajectory in the data set, effectively making about 6.4×10^6 trajectories. Fig. 2C is decomposed in Fig. 4 and, thus, uses the same data. In Fig. 3, we evenly split the two values of α among all trajectories. In Fig. 4B, C & D we use ATTM trajectories with $\sigma = 0.3$ and $\gamma = 0.4$ (see [4, 21], do not confuse with σ_{noise}). We simulate them randomly sampling D and the segment lengths accordingly, and the values here come from analysing the resulting trajectories, which have 18 different segments on average. For Fig. 4E, F & G we consider 755 experimental trajectories of the pathogen-recognition receptor DC-SIGN containing from 200 to 2000 frames sampled at 60 Hz. We obtain all the results in Fig. 5 from 4734 trajectories of the integrin $\alpha5\beta1$ containing from 20 to 500 frames sampled at 33 Hz. The data set for Fig. 2B is a sub-set of the one from Fig. 2A. In Fig. 2C, we use the same trajectories from Fig. 2A & B and add 128 random levels of localization noise to each of them, effectively making 6.144×10^6 noisy trajectories. In Fig. 2D, we average over all D values for each segment length to estimate the uncertainty. We take D in intervals of 0.2 within the given range (31 values), which combined with over 2×10^5 trajectories (over 7×10^5 segments combined) result in ~ 125 segments per D and length value.

Appendix C: Diffusion coefficient prediction

In the Results section, we study the capability of STEP to properly infer the diffusion coefficient at every time step and detect changes in diffusive behavior. Here, we complement the analysis presented in the main text by considering additional factors that impact the performance, such as the number of segments in the trajectories and the localization noise, typical of experimental setups. We show the results in Fig. 2.

We investigate the effect of the number of segments on the characterization of the trajectories. To test it, we fix the segment length and generate trajectories with one to eleven segments (zero to ten changepoints), resulting in trajectories with very different lengths (see Appendix B 3 for details). In Fig. 2A, we see a slight increase of the relative error with the number of segments, although it has a much lesser impact than the segment length, e.g., it is harder to characterize a single segment of twenty points than eight consecutive segments of 40 time steps. Importantly, even in the presence of 10 changepoints, STEP still heavily outperforms the TA-MSD approach applied to segments (no changes) of the same size, e.g., the whole curve for a segment length of 20 in Fig. 2A is well below the TA-MSD point for a segment length of 20 in Fig. 2C.

In Fig. 2E and F, we show how to combine STEP with a KCPD method to detect diffusion changes. In Fig. 2B, we show the performance as a function of the number of segments. We see that the shortest segments are the hardest to characterize. However, segment length becomes less important for sufficiently long ones, as the curves for lengths 40 and 60 behave fairly similarly. We see that STEP achieves a better

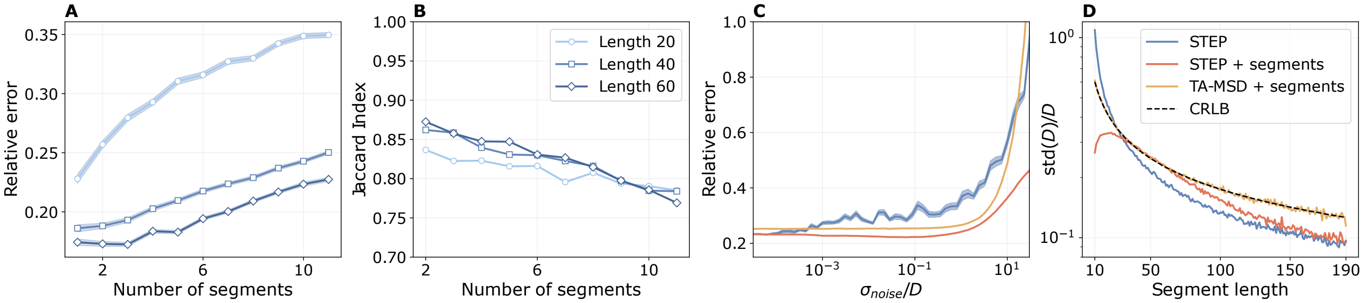

Figure 2. **STEP performance predicting the diffusion coefficient at every time step.** (**A**) Relative error of STEP as a function of the number of segments at three different segment lengths. (**B**) Prediction accuracy of STEP as a function of the number of segments at three different segment lengths. (**C**) Relative error of STEP (blue), STEP with known segments (red), and TA-MSD with known segments (yellow) as a function of the ratio between the localization noise's standard deviation σ and the diffusion coefficient D. (**D**) Prediction uncertainty as a function of the segment length for each of the previous methods compared to the Cramér-Rao lower bound (CRLB).

score for shorter segments when the trajectories are very long (11 segments). This suggests that every additional changepoint in the trajectory adds a similar amount of error sources which are eventually outweighed by the accumulated errors along the trajectory as it gets longer. Nonetheless, even in the most challenging cases with 11 segments, STEP correctly detect the vast majority of the points.

Thus, we study the resilience of our method to noise. In experimental scenarios, trajectories are affected by localization noise, which is usually modeled as Gaussian noise of variance σ^2_{noise} added to the trajectories. Since we consider diffusion coefficients at very different scales along the trajectories, in Fig. 2C, we plot the error as a function of the ratio between the noise's standard deviation and the diffusion coefficient. We see that STEP strongly outperforms the TA-MSD approach with known segments even well beyond the noise levels present in relevant experimental scenarios (usually $\sigma_{\text{noise}}/D < 10^{-1}$). Surprisingly, STEP can correctly extract the diffusion coefficient of constant segments (red line) even in the presence of large noise ($\sigma_{\text{noise}}/D > 10$).

Finally, we investigate STEP's ability to estimate D with higher precision that the TA-MSD approach (as shown in Fig. 2B), which is considered the optimal estimator for this calculation as documented by Michalet et al. [23]. To assess the prediction uncertainty of various methods, we examine it in relation to segment length in Fig. 2D. Our observations confirm that the TA-MSD fit is an optimal, unbiased estimator for D as it aligns closely with the Cramér-Rao lower bound (CRLB) (yellow and dashed black lines). Interestingly, STEP consistently deviates from the CRLB (blue and red lines below the dashed black line), suggesting a degree of bias in its estimation.

Several factors can contribute to introducing bias in the resulting model. For example, STEP estimates the logarithm of D, which can introduce bias since the use of logarithms typically leads to estimators with reduced variance. It is also essential to note that the model's accuracy heavily depends on both the training data and the loss function employed during training. In this specific case, the distribution of segment lengths in the training dataset follows an exponential function, resulting in a higher proportion of shorter segments. From the perspective of minimizing loss, there is a tendency to prioritize accuracy improvement for the shorter segments, even if it comes at the expense of accuracy for the longer ones.

Appendix D: Anomalous diffusion exponent prediction for various diffusion models

In the Results section, we briefly show how to use STEP to study particles that randomly switch between anomalous diffusing states. Here, we thoroughly characterize the suitability of the method for such task. We use STEP to predict the anomalous diffusion exponent α at every time step of trajectories composed of segments with constant anomalous diffusion exponent and diffusion model, as we detail in Appendix B 3.

In the main text, we have already studied how the mean absolute error (MAE) in the α prediction depends on the segment length, and we have compared STEP to two reference methods. As an additional reference, we obtain an MAE over all trajectories and time steps of 0.271 with STEP, 0.275 with STEP and known segments, 0.368 with TA-MSD and known segments, and CONDOR achieved an MAE of 0.237 for trajectories of the same lengths in the AnDi Challenge [3].

Furthermore, we can look at the performance segregated by the diffusion model. We report the MAE over all segments belonging to each anomalous diffusion model in Fig. 3A. We observe clear differences for some of the models with CTRW, FBM, and LW segments holding the lowest errors. Additionally, we provide a histogram of predicted and true α for all diffusion models in Fig. 4.

In particular, the MAE in scaled Brownian motion (SBM) segments is significantly larger than in the other

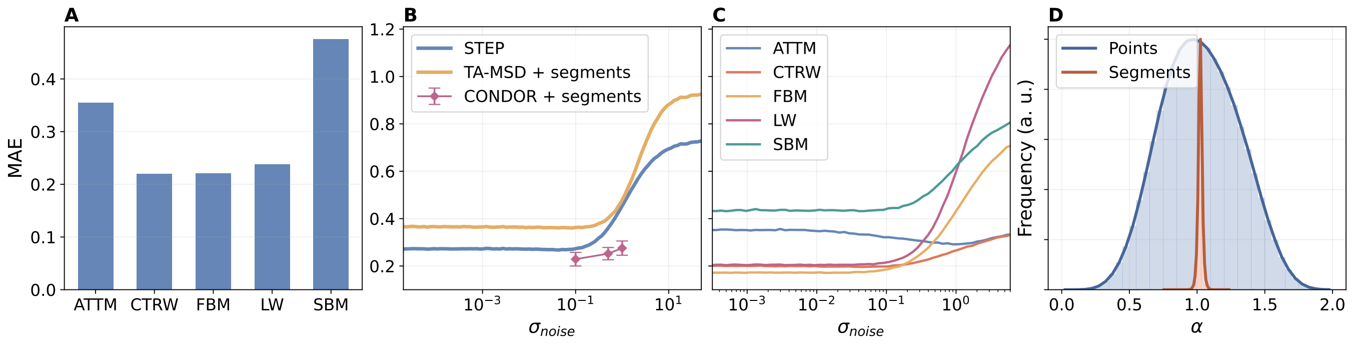

Figure 3. **STEP performance to predict α in terms of the mean absolute error (MAE).** We consider the anomalous diffusion models: ATTM, CTRW, FBM, LW and SBM (see Appendix B 3). (**A**) Prediction MAE by diffusion model. (**B**) MAE as a function of the localization noise for STEP, TA-MSD, and CONDOR, the latter two with known segments. (**C**) MAE as a function of the localization noise for STEP separated by the diffusion model. The blue line in (**B**) corresponds to the average of the lines presented in this panel. (**D**) Prediction of α for Brownian motion trajectories. The blue distribution shows the pointwise prediction for the trajectories, while the red one shows the mean prediction over trajectories. The distributions have been normalized to have the same maximum value.

models. This has already been observed in previous works (see for instance Fig. 2d of Ref. [3]), although the differences here are larger. A detailed inspection shows that the biggest errors come from shorter segments, in agreement with the results from Fig. 2D. This is reasonable since the aging in SBM is the source of the anomalous diffusion [1] and therefore it requires longer segments to be correctly characterized. It is also reasonable to expect the largest errors to happen whenever $\alpha \in (0, 2)$ is close to its range limits and the predictions are in the opposite side, which is mitigated in models with restricted ranges of α.

STEP displays a clear tendency to predict $\alpha \sim 0.8$ for SBM segments, as we can see in the right-most column of Fig. 4. This behavior is enhanced by the presence of noise, suggesting that the model struggles to identify any clear behavior in short segments, which also happen to be the most common.

Interestingly, we find a similar trend in CTRW segments, where the model has a tendency to predict $\alpha \simeq 0.25$, corresponding to nearly immobile particles. CTRW trajectories are characterized by jumps at random times, resulting in segments in which the particle does not move, usually referred to as waiting times. Hence, many CTRW segments in our heterogeneous trajectories do not display any movement due to their short lengths, corresponding to a waiting time section. Therefore, it is impossible for the model to correctly predict α, as it does not have any information to work with.

To a lesser extent, we also find that the model predicts $\alpha \sim 1$ for low anomalous diffusion exponents in ATTM segments. In ATTM trajectories with small α, we encounter very long segments with low diffusion coefficients. Similar to the CTRW case, we encounter parts of these long segments in our heterogeneous trajectories containing a unique diffusion coefficient, thus behaving like Brownian motion along the observed time window. Hence, the predictions $\alpha \sim 1$ are correct in these cases.

We proceed to study the resilience of the methods to localization noise, as we do in Appendix C. We present the MAE as a function of the noise standard deviation σ_{noise} in Fig. 3C. We observe a consistent performance of all the methods until reaching considerable levels of noise. Again, STEP is comparable to CONDOR despite the latter having the advantage of knowing the segments beforehand.

As we have seen throughout this section, characterizing some diffusion models is harder than others and the localization noise has a different impact on them, as we show in Fig. 3D and Fig. 4. While increasing the noise level has an overall negative effect, we see that the performance on LW segments suffers the most, while the performance on CTRW segments is barely affected. Overall, the errors start to increase significantly beyond $\sigma_{\text{noise}} \sim 2 \times 10^{-1}$, which would correspond to harsh experimental conditions. Interestingly, ATTM segments see a drop in MAE with increasing noise for a limited range.

In conclusion, we analyze the predictions of α for Brownian motion trajectories featuring random diffusion changes, as illustrated inFig. 2A & B. The pointwise prediction (blue distribution) averages to $\alpha = 1.02$, which aligns closely with the expected value of 1. This outcome gains further credibility when we compute the average predicted α across the entire trajectory (red distribution), since it shows that all trajectories are consistently predicted to have α values approximating 1. Although these trajectories closely resemble those of ATTM, characterized by random changes in diffusion coefficients, our model correctly discerns that the trajectories in question do not exhibit anomalous diffusion. This determination is based on the fact that the diffusion coefficients and dwell times do not satisfy the conditions outlined in Fig. 4 and related text, which are necessary to induce anomalous behavior.

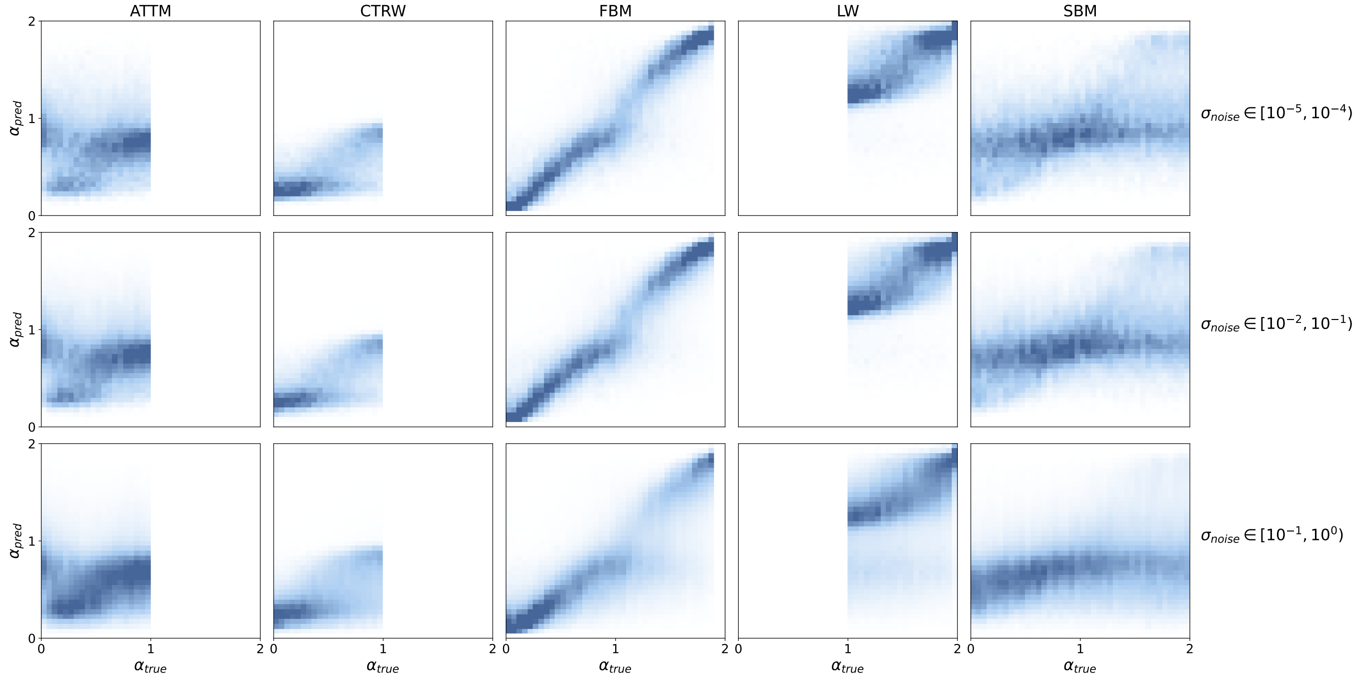

Figure 4. **Predicted vs true anomalous diffusion exponent.** 2D histograms showing the true and predicted anomalous diffusion exponents for the different diffusion models. Each column contains the histograms belonging to a different diffusion model for three different localization noise levels: $\sigma_{\text{noise}} \in [-5, -4)$ (top), $\sigma_{\text{noise}} \in [-2, -1)$ (middle), and $\sigma_{\text{noise}} \in [-1, 0)$ (bottom). The low-noise histograms (top row) result in Fig. 2C when combined together.

Appendix E: Experimental materials and methods

1. Cell culture and plating

For the live-cell single-molecule imaging experiments involving the integrin $\alpha5\beta1$, HeLa cells were cultured in DMEM (Gibco, 11960-044), supplemented with 10% (v/v) fetal bovine serum (FBS, Sigma). Cells were tested for mycoplasma contamination using PCR (Biotools kit, 4542). For fluorescence imaging, glass-bottom dishes (IBIDI, 81158) were coated with fibronectin (FN, Sigma, F2008) by placing 10 μg/mL FN on the glass for 1 h at 37 °C, and then blocked with BSA 2 mg/mL for 1 h at 37 °C. Cells were plated at a density of 5×10^4 cell/dish and cultured for 24 h prior to use.

2. Preparation of half-antibody fragments

Half-antibody fragments were obtained following a protocol similar to the one used in [24]. Briefly, mouse anti-human integrin $\alpha5$ antibody (50 μL; BD Biosciences, 610633) was dialyzed (ThermoFisher, Slide-A-Lyze MINI Dialysis Device, 2K) against PBS overnight at room temperature to replace the commercial buffer. Then, antibodies were reduced with 1 mM DTT for 30 min at room temperature and dialyzed again, using Slide-A-Lyze MINI Dialysis Device 2K, for 4 h at room temperature against PBS to remove DTT. To avoid reassociation of reduced antibodies, sulfhydryl groups were blocked by incubating with iodoacetamide 20 mM for 1 h at 4 °C with agitation. Iodoacetamide was then removed from the reaction by dialysis overnight at 4 °C. Finally, reduced antibodies were biotinylated with a 10-fold molar excess of EZ-Link Sulfo-NHS_LC_Biotin (Thermo Scientific) for 30 min at room temperature with agitation and stored at 4 °C until use.

3. Single-molecule labeling

Biotinylated half-antibody fragments were conjugated to streptavidin-coated quantum dots (QD655 streptavidin conjugate, Invitrogen, Q10123mp). Cells were washed 3 times with washing buffer (PBS with 6% BSA) and labeled with half antibody-quantum dots (about 1 nM) in washing buffer (200 μL per dish) for 15 min at 37 °C, followed by two washes.

4. Live-cell single-molecule imaging

Imaging was performed using a Leica DMi8 fluorescence microscope. Samples were illuminated in total internal reflection fluorescence (TIRF) geometry. Excitation was achieved with a CW laser (Obis, Coherent, λ=488 nm, <1 kW/cm^2). Fluorescence was recorded using an oil-immersion objective (Leica, 100X,

NA=1.47) and an sCMOS camera (Photometrics 95B) with appropriate filters (Chroma). Movies were recorded at a frame rate of 33 Hz. A microscope environment chamber (Okolab) was used to keep cells in a 5% CO_2 atmosphere while recording.

5. Single-particle tracking

Particle detection and tracking were performed using u-track [25]. The detection (Gaussian Mixture-Model Fitting) and tracking parameters were optimized based on visual inspection and performance diagnostic of the resulting detection and tracking. All image and data analysis tasks were performed in MATLAB 2020a and more recent versions (The MathWorks, Natick, MA). Videos were loaded into MATLAB using Bio-Formats [26].

[1] Ralf Metzler, Jae-Hyung Jeon, Andrey G Cherstvy, and Eli Barkai. Anomalous diffusion models and their properties: non-stationarity, non-ergodicity, and ageing at the centenary of single particle tracking. *Physical Chemistry Chemical Physics*, 16(44):24128–24164, 2014.

[2] Joseph Klafter and Igor M Sokolov. *First steps in random walks: from tools to applications*. OUP Oxford, 2011.

[3] Gorka Muñoz-Gil, Giovanni Volpe, Miguel Angel Garcia-March, Erez Aghion, Aykut Argun, Chang Beom Hong, Tom Bland, Stefano Bo, J Alberto Conejero, Nicolás Firbas, et al. Objective comparison of methods to decode anomalous diffusion. *Nature Communications*, 12:6253, 2021.

[4] Pietro Massignan, Carlo Manzo, Juan A Torreno-Pina, Maria F García-Parajo, Maciej Lewenstein, and GJ Lapeyre Jr. Nonergodic subdiffusion from brownian motion in an inhomogeneous medium. *Physical Review Letters*, 112(15):150603, 2014.

[5] Harvey Scher and Elliott W. Montroll. Anomalous transit-time dispersion in amorphous solids. *Phys. Rev. B*, 12:2455–2477, Sep 1975.

[6] Benoit B. Mandelbrot and John W. Van Ness. Fractional brownian motions, fractional noises and applications. *SIAM Review*, 10(4):422–437, 1968.

[7] J. Klafter and G. Zumofen. Lévy statistics in a hamiltonian system. *Phys. Rev. E*, 49:4873–4877, Jun 1994.

[8] S. C. Lim and S. V. Muniandy. Self-similar gaussian processes for modeling anomalous diffusion. *Phys. Rev. E*, 66:021114, Aug 2002.

[9] Borja Requena and Gorka Muñoz-Gil. Step python library (https://github.com/borjarequena/step), December 2022.

[10] Adam Paszke, Sam Gross, Francisco Massa, Adam Lerer, James Bradbury, Gregory Chanan, Trevor Killeen, Zeming Lin, Natalia Gimelshein, Luca Antiga, Alban Desmaison, Andreas Kopf, Edward Yang, Zachary DeVito, Martin Raison, Alykhan Tejani, Sasank Chilamkurthy, Benoit Steiner, Lu Fang, Junjie Bai, and Soumith Chintala. Pytorch: An imperative style, high-performance deep learning library. In *Advances in Neural Information Processing Systems*, volume 32. Curran Associates, Inc., 2019.

[11] Jeremy Howard and Sylvain Gugger. Fastai: A layered api for deep learning. *Information*, 11(2), 2020.

[12] Charles Truong, Laurent Oudre, and Nicolas Vayatis. Selective review of offline change point detection methods. *Signal Processing*, 167:107299, 2020.

[13] Tong He, Zhi Zhang, Hang Zhang, Zhongyue Zhang, Junyuan Xie, and Mu Li. Bag of tricks for image classification with convolutional neural networks. In *Proceedings of the IEEE/CVF Conference on Computer Vision and Pattern Recognition*, pages 558–567, 2019.

[14] Ashish Vaswani, Noam Shazeer, Niki Parmar, Jakob Uszkoreit, Llion Jones, Aidan N Gomez, Łukasz Kaiser, and Illia Polosukhin. Attention is all you need. In *Advances in Neural Information Processing Systems*, volume 30, 2017.

[15] Diederik P. Kingma and Jimmy Ba. Adam: A method for stochastic optimization. In *ICLR (Poster)*, 2015.

[16] Leslie N. Smith. A disciplined approach to neural network hyper-parameters: Part 1 – learning rate, batch size, momentum, and weight decay. *arXiv preprint arXiv:1803.09820*, 2018.

[17] Leslie N. Smith and Nicholay Topin. Super-convergence: very fast training of neural networks using large learning rates. In *Artificial Intelligence and Machine Learning for Multi-Domain Operations Applications*, volume 11006, pages 369 – 386. International Society for Optics and Photonics, SPIE, 2019.

[18] Geoffrey E. Hinton, Nitish Srivastava, Alex Krizhevsky, Ilya Sutskever, and Ruslan R. Salakhutdinov. Improving neural networks by preventing co-adaptation of feature detectors. *arXiv preprint arXiv:1207.0580*, 2012.

[19] Stephen Hanson and Lorien Pratt. Comparing biases for minimal network construction with back-propagation. In *Advances in Neural Information Processing Systems*, volume 1, 1988.

[20] Anders Krogh and John Hertz. A simple weight decay can improve generalization. In *Advances in Neural Information Processing Systems*, volume 4, 1991.

[21] Carlo Manzo, Juan A Torreno-Pina, Pietro Massignan, Gerald J Lapeyre Jr, Maciej Lewenstein, and Maria F Garcia Parajo. Weak ergodicity breaking of receptor motion in living cells stemming from random diffusivity. *Physical Review X*, 5(1):011021, 2015.

[22] Gorka Muñoz-Gil, Borja Requena, Giovanni Volpe, Miguel Angel Garcia-March, and Carlo Manzo. Andi_datasets python library (github.com/andichallenge/andi_datasets), 2021.

[23] Xavier Michalet and Andrew J Berglund. Optimal diffusion coefficient estimation in single-particle tracking. *Physical Review E*, 85(6):061916, 2012.

[24] Shalini T Low-Nam, Keith A Lidke, Patrick J Cutler, Rob C Roovers, Paul MP van Bergen en Henegouwen, Bridget S Wilson, and Diane S Lidke. Erbb1 dimerization is promoted by domain co-confinement and stabilized by ligand binding. *Nature Structural & Molecular Biology*, 18(11):1244–1249, 2011.

[25] Khuloud Jaqaman, Dinah Loerke, Marcel Mettlen, Hirotaka Kuwata, Sergio Grinstein, Sandra L Schmid, and Gaudenz Danuser. Robust single-particle tracking in live-cell time-lapse sequences. *Nature Methods*, 5(8):695–702, 2008.

[26] Melissa Linkert, Curtis T Rueden, Chris Allan, Jean-Marie Burel, Will Moore, Andrew Patterson, Brian Loranger, Josh Moore, Carlos Neves, Donald MacDonald, et al. Metadata matters: access to image data in the real world. *Journal of Cell Biology*, 189(5):777–782, 2010.
