# Supplementary material for: Inferring pointwise diffusion properties of single trajectories with deep learning
Source: Biophys J. 2023 Oct 17;122(22):4360–9. doi: 10.1016/j.bpj.2023.10.015 (PMC10698275; doi:10.1016/j.bpj.2023.10.015)
Supplement: Document S2. Article plus supporting material [file mmc2.pdf]

Biophysical Journal
Article

Biophysical Society

Inferring pointwise diffusion properties of single trajectories with deep learning

Borja Requena,[1] Sergi Masó-Orriols,[2,5] Joan Bertran,[2,5] Maciej Lewenstein,[1,3] Carlo Manzo,[2,5,*] and Gorka Muñoz-Gil[4,*]
[1]ICFO – Institut de Ciències Fotòniques, The Barcelona Institute of Science and Technology, Castelldefels (Barcelona), Spain; [2]Facultat de Ciències, Tecnologia I Enginyeries, Universitat de Vic – Universitat Central de Catalunya (UVic-UCC), Vic, Spain; [3]ICREA, Pg. Lluís Companys 23, Barcelona, Spain; [4]Institute for Theoretical Physics, University of Innsbruck, Innsbruck, Austria; and [5]Institut de Recerca i Innovació en Ciències de la Vida i de la Salut a la Catalunya Central (IRIS-CC), Vic, Barcelona, Spain

ABSTRACT To characterize the mechanisms governing the diffusion of particles in biological scenarios, it is essential to accurately determine their diffusive properties. To do so, we propose a machine-learning method to characterize diffusion processes with time-dependent properties at the experimental time resolution. Our approach operates at the single-trajectory level predicting the properties of interest, such as the diffusion coefficient or the anomalous diffusion exponent, at every time step of the trajectory. In this way, changes in the diffusive properties occurring along the trajectory emerge naturally in the prediction and thus allow the characterization without any prior knowledge or assumption about the system. We first benchmark the method on synthetic trajectories simulated under several conditions. We show that our approach can successfully characterize both abrupt and continuous changes in the diffusion coefficient or the anomalous diffusion exponent. Finally, we leverage the method to analyze experiments of single-molecule diffusion of two membrane proteins in living cells: the pathogen-recognition receptor DC-SIGN and the integrin $\alpha5\beta1$. The analysis allows us to characterize physical parameters and diffusive states with unprecedented accuracy, shedding new light on the underlying mechanisms.

SIGNIFICANCE Understanding the diffusion of particles in biological systems is crucial to unravel fundamental mechanisms in various fields of study. This research introduces a machine-learning method able to predict key physical properties, such as the diffusion coefficient and anomalous diffusion exponent, at each time step of the input trajectory. The method is especially well suited to characterize processes with either discrete or continuous diffusive changes without any prior information about the process. We validate it using synthetic trajectories and subsequently apply it to study the diffusion of membrane proteins in living cells. The findings provide unprecedented accuracy in the extraction of physical parameters and diffusive states, offering valuable insights into the underlying physical mechanisms of biological processes.

INTRODUCTION

Advances in optical imaging have made it possible to observe single molecules in living biological systems (1). When combined with particle tracking algorithms, these techniques allow tracing the movement of individual molecules, viruses, and organelles with nanometric precision, enabling the study of transport mechanisms in complex biological environments. Through the biophysical characterization of trajectories, we can extract meaningful parameters to describe physical and biological processes, such as nanoscopic particles in the cell (2), active particles in complex environments (3), or even the motion of wild animals (4). However, accurately quantifying the trajectories remains a challenging task due to their stochastic nature and to experimental drawbacks, such as imaging noise and the emitter's photophysics (5).

Nowadays, there exists a plethora of methods to characterize diffusive processes. The most common approaches are based on the calculation of the mean squared displacement (MSD; see Appendix A). Fitting the MSD offers an optimal way to estimate the diffusion coefficient from trajectories undergoing Brownian motion in many circumstances (6,7). However, studying anomalous diffusion trajectories is more challenging due to the intricate properties of the system's dynamics, e.g., nonergodicity or aging

Submitted May 25, 2023, and accepted for publication October 13, 2023.
*Correspondence: carlo.manzo@uvic.cat or munoz.gil.gorka@gmail.com
Editor: Gerhard Schutz.
https://doi.org/10.1016/j.bpj.2023.10.015
© 2023 Biophysical Society.
This is an open access article under the CC BY license (http://creativecommons.org/licenses/by/4.0/).

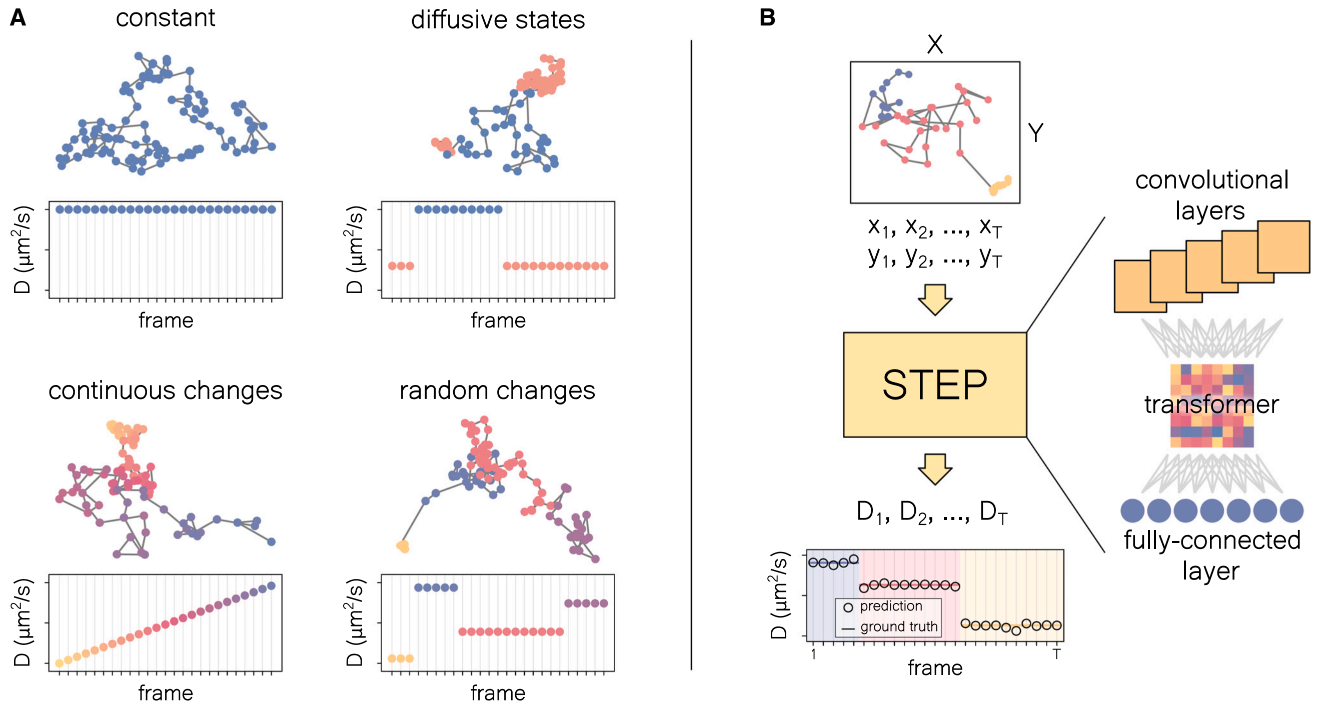

FIGURE 1 Heterogeneous trajectories and the STEP pipeline. (*A*) Examples of trajectories and the corresponding diffusion coefficient *D* as a function of time for constant *D*, changes within a discrete set of states with fixed *D*, continuous and monotonous change of *D*, and switch between random *D*s. (*B*) Schematic of the pipeline of STEP: an input trajectory is fed to the architecture, which consists of a stack of convolutional layers, a transformer encoder, and a pointwise feedforward layer. The model's output is the pointwise prediction of the diffusion parameter of interest (in this case *D*). To see this figure in color, go online.

(8), and artifacts associated to the presence of experimental noise (9). The anomalous diffusion exponent α can be faithfully extracted from the fit of the MSD averaged over an ensemble of trajectories (10). When dealing with ensembles of heterogeneous diffusers, individual information can be obtained by the time-averaged MSD (TA-MSD) for ergodic and sufficiently long trajectories (11). Additionally, when dealing with some specific diffusion models, we can exploit this knowledge to use methods based, for instance, on the power spectral density of single trajectories (12,13) or the Bayesian estimation of diffusive properties (14). Recently, as we extensively discuss below, machine-learning-based approaches (15) have been shown to boost these analyses.

Nonetheless, the methods described above can only be used to study trajectories with constant diffusive properties. In cellular systems, a widespread diffusion feature is the occurrence of time-dependent changes of motion (16). Typically, these changes are associated with transient interactions with other components (17–19) resulting in the sudden variation of a parameter, e.g., the diffusion coefficient, which can switch between a discrete (20) and continuous set of levels (21–23). Furthermore, they can induce smooth changes such as those associated with the spatiotemporal heterogeneity of the environment (24). Examples of trajectories undergoing this kind of diffusion are schematically depicted in Fig. 1 *A*.

Trajectories with time-dependent diffusion properties pose an additional challenge to characterize the motion of individual particles, which has been tackled with different approaches. For trajectories displaying abrupt changes, the combination of statistical methods with segmentation algorithms (16,25,26) is a valuable strategy but cannot deal with long-range correlations and often offers limited time resolution due to temporal averaging. On the other hand, model-dependent methods such as the hidden Markov model have been quite successful in describing heterogeneous diffusion (27–29), although they require prior knowledge about the diffusive states involved and their kinetic scheme. Recently, data-driven approaches have shown remarkable capabilities to extract information from individual stochastic trajectories, even in the presence of changes of diffusion properties (15,30–32).

In this work, we propose STEP, a method based on state-of-the-art deep-learning architectures to extract pointwise diffusion features from individual trajectories without any prior information (see Fig. 1 *B*). This allows STEP to overcome many of the presented methods' limitations, making it suitable for a wide range of applications. STEP features the most recent advances in sequence-to-sequence learning (33), which have shown impressive results in natural language processing tasks and beyond (34–36). It combines convolutional (37) and attention layers (38) to cope with the presence of short- and long-range correlations,

providing remarkable performance over trajectories of any length and in the presence of noise.

The article is structured as follows: first, we introduce the main aspects of the proposed method. Then, we show the ability of STEP to predict diffusion properties, such as the diffusion coefficient and the anomalous diffusion exponent, on simulated data reproducing experimentally relevant scenarios. Then, we analyze simulated trajectories with smoothly varying diffusion coefficients, showing that STEP correctly finds the expected scaling. Finally, we use STEP to study two experimental datasets obtained by the tracking of single-molecule live-cell imaging experiments and reporting the motion of two membrane receptors: 1) the pathogen-recognition receptor DC-SIGN, which has been associated with random changes of diffusion coefficients (23), and 2) the $\alpha 5\beta 1$ integrin, expected to be transiently arrested by binding to the cytoskeleton and the extracellular matrix (39). We conclude with a discussion of the results.

MATERIALS AND METHODS

The STEP architecture

Recently, we have witnessed an enormous effort in the development of deep-learning approaches to study diffusive processes (15). Previous works usually focused on characterizing diffusive properties of single trajectories (i.e., predicting an overall or average diffusive parameter for each input trajectory (40–44)). Recent works have proved the suitability of this approach to study complex phenomena in different experimental scenarios (45,46).

With STEP, we propose a sequence-to-sequence approach (33) that translates position coordinates into the diffusion properties of interest at every time step of the input trajectory, as illustrated in Fig. 1 *B*. In this way, the input and the output of the model have the same length. Although it is effectively impossible to characterize diffusion from a single displacement due to its stochastic nature, STEP uses the whole trajectory as context to perform the prediction at every point.

This approach allows us to study trajectories whose diffusion properties can vary over time with different patterns: from trajectories with constant diffusive properties to trajectories that sharply switch between different diffusive states, or with diffusive parameters that change continuously over time (see Fig. 1 *A* for examples). Unlike previous works, where expert input is needed to choose an appropriate method, STEP can be seamlessly applied to any diffusive data. Importantly, it does not rely on prior assumptions, such as the number of changepoints (47,48) or the properties of the expected diffusive states (49).

In diffusion phenomena, we deal with complex statistical signals that can exhibit various types of time correlations. Furthermore, we often encounter trajectories with very different lengths, even in the same experiment. Hence, it is crucial that the machine-learning models are length independent and able to capture correlations at different timescales to ensure that they are as applicable as possible. State-of-the-art architectures for diffusion characterization rely on very different approaches (50–52). Several models achieve outstanding performance by combining recurrent neural networks that account for long-range correlations (42,48) with convolutional neural networks that capture local features (46,53,54).

We propose an architecture combining convolutional and self-attention mechanisms. Interestingly, very recent works have shown analogous strategies, both with supervised (31) and unsupervised approaches (55). First, the input trajectory is processed by a series of convolutional layers that we build after the XResNet architecture (56). Then, the result follows through a transformer encoder (38), which can capture global correlations. Finally, we use a pointwise fully connected layer of nonlinear neurons to obtain the desired output dimension, as we illustrate in Fig. 1 *B*.

To produce the results presented in this work, we train two models: one to infer the diffusion coefficient D and another for the anomalous diffusion exponent α. Each is trained on simulated noisy trajectories that present abrupt changes in their respective diffusion properties (D or α). Throughout this work, we mainly consider two-dimensional (2D) trajectories, but the whole method can be easily adapted to any dimensions.

We provide a detailed description of the architecture, its training procedure, and the data in Appendix B. We also provide a library in Ref. (57) containing the code and extensive tutorials to reproduce the results of this work.

Baselines

STEP provides pointwise diffusion properties for input trajectories, which, to the best of our knowledge, is a distinct task from that addressed by any existing method. In this context, it is challenging to perform a straightforward and equitable comparison of STEP with these methods, particularly considering that typical approaches involve the combination of various methods to achieve similar results.

Thus, instead of creating complex benchmarks, we compare STEP to the best alternatives proposed so far for the extraction of diffusion properties such as D and α. Since these methods cannot deal with time-dependent changes of diffusion, we apply them to trajectories pre-segmented according to the ground truth. For calculating D from Brownian trajectories, we employ the fitting of the TA-MSD, which is the optimal estimator for D in most cases (6). For estimating α from trajectories undergoing anomalous diffusion, we employ the TA-MSD fit in logarithmic space and CONDOR (47), recognized as the leading approach for this task in the AnDi Challenge (15). Although these methods benefit from a significant advantage due to the pre-segmentation, STEP typically achieves comparable performance (see Fig. 2).

RESULTS

Pointwise prediction of diffusion properties

We first validate STEP on the task of inferring the pointwise diffusion coefficient from simulated trajectories reproducing transient Brownian motion with abrupt changes of diffusion coefficient. The diffusion coefficient can randomly vary in the range $D \in [10^{-3}, 10^3]$ and the dwell time in each diffusion coefficient is drawn from an exponential distribution between 10 and 190 (mean 57) time steps. Further details of the simulations and the datasets are described in Appendix B3. A 2D histogram of the ground truth versus the predicted diffusion coefficient shows that STEP can precisely determine the diffusion coefficient across its whole range (Fig. 2 *A*) with an overall relative error $|D_{\mathrm{true}} - D_{\mathrm{pred}}|/D_{\mathrm{true}} = 0.226$.

To further explore the performance of STEP, we calculate the relative error as a function of the segment length (i.e., the dwell time for each D; blue line in Fig. 2 *B*). The error increases for shorter segments, meaning that STEP needs sufficient statistics from surrounding points with similar properties to accurately predict the diffusive properties of a given point. Comparing these results with the TA-MSD's prediction on pre-segmented trajectories, STEP is close to the optimal target performance (blue vs. yellow lines in Fig. 2 *B*). Notably, when STEP is provided with

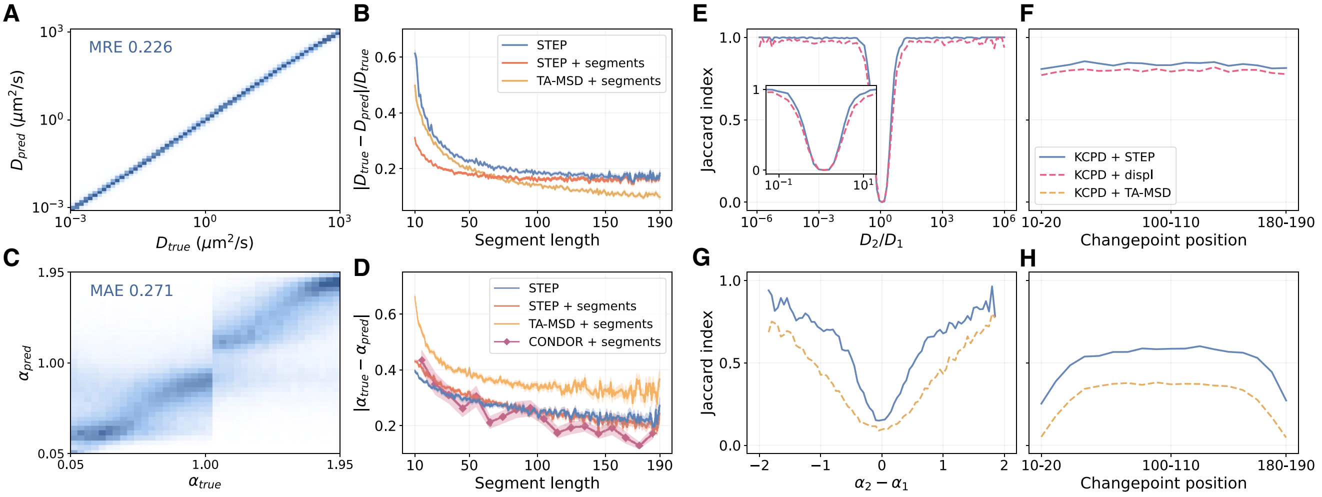

FIGURE 2 Time-dependent diffusion properties prediction. (*A*) 2D histogram of the predicted diffusion coefficient D compared to the ground truth. The mean relative error (MRE) over the whole test set is 0.226. (*B*) Relative error for the prediction of D as a function of the segment length. (*C*) 2D histogram of the predicted anomalous diffusion exponent α compared to the ground truth. The MAE over the whole test set is 0.271. (*D*) MAE for the prediction of α as function of the segment length. (*E–H*) Jaccard index for the changepoint detection problem as a function of (*E*) the ratio between consecutive segment Ds, (*F*) the changepoint position for D, (*G*) the difference between consecutive segment αs, and (*H*) the changepoint position for α. For details about the data used in each panel, see Appendix B3 and Table I therein. To see this figure in color, go online.

pre-segmented trajectories, we observe a further improvement, with a nearly twofold reduction of the error at short segment lengths (red line in Fig. 2 *B*), demonstrating outstanding prediction capabilities. However, it is outperformed by the TA-MSD baseline in long segments. We provide a deeper analysis about this performance trade-off in Appendix C, including a comparison to the Cramér-Rao lower bound (6).

We then examine the ability of STEP to predict the anomalous diffusion exponent α. We consider trajectories composed of segments following the same length distribution as in the Brownian motion case. Each segment is simulated using a different anomalous diffusion model and $\alpha \in [0.05, 2]$ (see Appendix B3). The 2D histogram of the ground truth vs. the predicted α in Fig. 2 *C* shows that STEP successfully predicts the anomalous diffusion exponent. We obtain a mean absolute error (MAE) $|\alpha_{\text{true}} - \alpha_{\text{pred}}| = 0.271$, which is in line with the top-scoring approaches for this task (48,53) in the anomalous diffusion challenge (15). As with most methods in the challenge, STEP is prone to errors for $\alpha \sim 1$ (15). In addition, since several models are inherently only sub- or super-diffusive and the method tends to predict values of α within the training range, we observe a discontinuous behavior in the histogram for $\alpha \sim 1$ (see Appendix A).

In Fig. 2 *D*, we report the MAE for α as a function of the segment length. STEP strongly outperforms the TA-MSD approach and shows a performance comparable to CONDOR. For this task, providing pre-segmented data to STEP marginally improves its performance for long segments, whereas it even reduces it for short ones. This result suggests that segment length is more important than the exact knowledge of the segment edges and STEP effectively combines local and global information.

In Appendix C and D, we extend the assessment of the performance of STEP as a function of the localization precision, the dwell-time duration, the number of segments in the trajectories, and the underlying diffusion model, showing that the method can be efficiently applied in a wide range of experimental conditions. Furthermore, we show that STEP correctly predicts $\alpha = 1$ for trajectories undergoing Brownian motion.

Detecting diffusive changepoints in heterogeneous trajectories

For trajectories undergoing sudden changes of diffusion properties, the exact knowledge of the points at which these changes occur is crucial to infer temporal properties and kinetic rates of the system and fully characterizing the underlying physical process. Although STEP does not explicitly detect changepoints, its output provides a precise estimation of the diffusion property that is supposed to change, hence simplifying the task of changepoint detection and location with respect to the use of raw data. To highlight this capability, we compare the results obtained by a state-of-the-art kernel changepoint detection (KCPD) method (58,59) when applied to STEP's predictions and to the time series of trajectory displacements. To assess the performance of the methods, we compute the Jaccard index (JI) considering as a true positive any changepoint predictions lying within a threshold distance $\mathcal{E}$ from the corresponding ground truth.

We first quantify the performance of the method to detect changes of the diffusion coefficient in heterogeneous

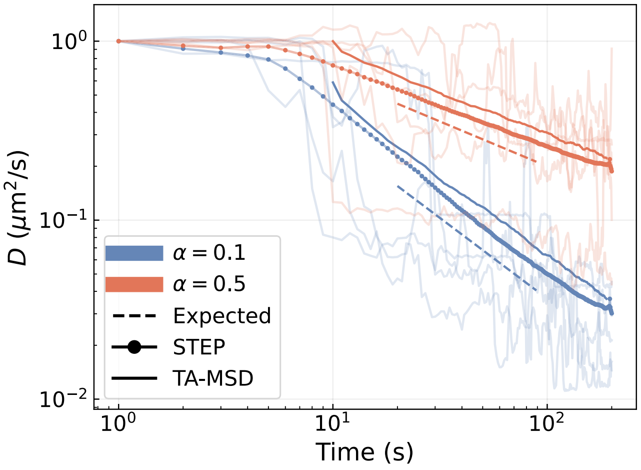

FIGURE 3 Continuous changes of diffusion properties. Predictions of the time-dependent diffusion coefficient of two sets of SBM trajectories with $\alpha = 0.1$ (blue) and 0.5 (red). The bold continuous lines show the average prediction over 3000 trajectories with STEP and a linear fit of the TA-MSD over a sliding window of 20 points. The thin continuous lines show a few example STEP predictions. The dashed lines indicate the theoretically expected scaling for every α. The lines have been normalized and shifted to compare their slopes easily. To see this figure in color, go online.

Brownian motion trajectories. We use a benchmark dataset with trajectories of 200 time steps exhibiting a single changepoint and set $\mathcal{E} = 5$. Applying the KCPD algorithm on the prediction of STEP, we can successfully detect the changepoints with high accuracy. The detection improves as the differences between consecutive segments increase, achieving a nearly perfect detection for segments whose diffusion coefficients are just one order of magnitude apart, as we show in Fig. 2 *E* (blue line). Furthermore, the method is robust with respect to the changepoint position within the trace (Fig. 2 *F*, blue line). In contrast, when we apply KCPD directly over the trajectory displacements (dashed purple lines) we observe a decrease in performance over the whole range of diffusion coefficient ratios. On average, STEP produces a 20% reduction in error, reaching an average JI of 0.833 compared to 0.796 obtained with the raw displacements.

We perform a similar analysis to detect changes in the anomalous diffusion exponent. To ease the analysis, we consider only trajectories undergoing fractional Brownian motion (60). Since the anomalous diffusion exponent is an asymptotic property that cannot be easily calculated from the raw data, to build our baseline, we compute α with a linear fit of the TA-MSD on a log-log scale using a sliding window of 30 time steps, which we then feed into the KCPD algorithm (dashed yellow lines). Expectedly, the larger the differences between segment parameters, the better we can detect the changepoints, as we show in Fig. 2 *G*. We obtain a 30% error reduction by using STEP with respect to the baseline method, achieving an average JI of 0.515 and 0.297, respectively, with $\mathcal{E} = 20$. However, these metrics show that finding changes in α is significantly harder than in D. Moreover, we also observe a performance drop when the changepoints are near the trajectory edges (Fig. 2 *H*). In these cases, we deal with short segments whose anomalous diffusion exponent can be hard to determine, as they rely on the arising of long-range correlations.

Revealing continuous changes of diffusion properties

When considering heterogeneous trajectories in the biological context, the typical behavior one expects is represented by particles undergoing diffusion with piecewise constant properties that can suddenly change, e.g., as the result of specific interactions with other biological components. However, the presence of molecular crowding and gradients of concentration can produce a continuous variation of diffusion properties over time. These changes might be challenging to detect due to the limited spatio-temporal resolution of the experiments or the lack of specific approaches for trajectory analysis. Since STEP predicts pointwise diffusion properties in a model-free fashion, it inherently features the capability to perform this kind of analysis, even without dedicated training.

To evaluate the performance of STEP on smoothly varying trajectories, we rely on simulations of scaled Brownian motion (SBM) (24). SBM trajectories are characterized by a time-dependent diffusion coefficient with a power-law relationship $D(t) \sim t^{\alpha - 1}$, where α is the anomalous diffusion exponent. Further details about simulations are given in Appendix B3.

In Fig. 3, we show the predictions obtained for the diffusion coefficient at every time step of trajectories with $\alpha = 0.1$ and 0.5. The shaded lines represent the STEP predictions obtained for individual trajectories that, despite the fluctuations, already indicate the decreasing trend. Averaging over trajectories (round marker) reveals the correct power-law scaling (dashed lines). We also obtain the correct scaling with a linear fit of the TA-MSD on a sliding window of 20 points over the trajectories (solid lines). As shown, STEP can capture the scaling earlier, since it is not limited by the size of the window. Furthermore, the inference of α correctly provides a nearly constant value throughout the trajectory, as expected.

Characterizing anomalous diffusion from changes of normal diffusive properties

To test the potential of STEP for the analysis of experimental trajectories, we use it to study the motion of the pathogen-recognition receptor DC-SIGN expressed in Chinese hamster ovarian cells (61). Previous analysis of these experiments revealed the occurrence of anomalous diffusion and weak ergodicity breaking as a consequence of stochastic changes of diffusion coefficient (23). This behavior was described in the framework of the annealed transit-time model (ATTM) (62), whose main features are schematically summarized in Fig. 4 *A*.

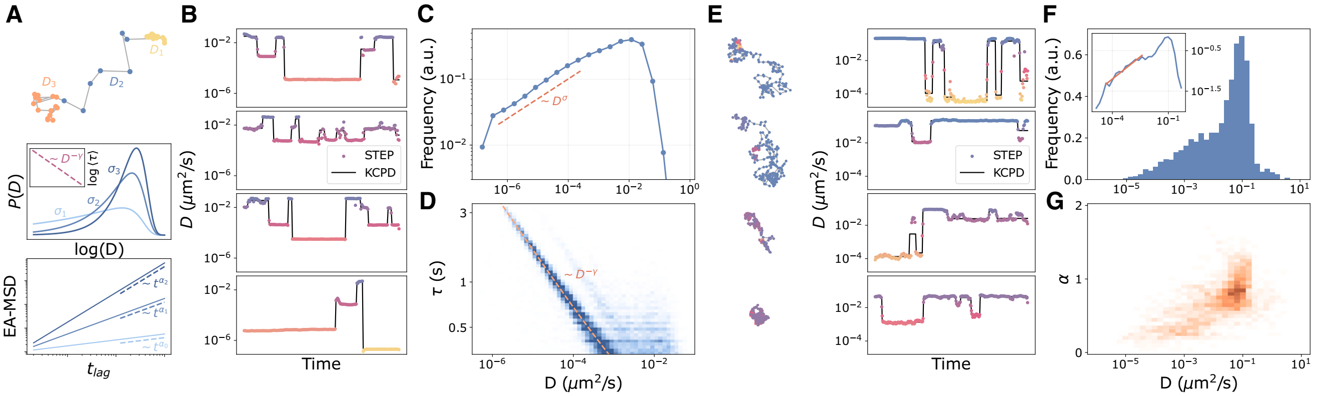

FIGURE 4 Switch between random diffusive states of the pathogen-recognition receptor DC-SIGN. (*A*) Characteristic features of the ATTM model: an exemplary trajectory undergoing changes of diffusion coefficient; a few examples of the distribution of D with different σ_i, and an example relation between the diffusion coefficient and the dwell time τ for a fixed γ; and the ensemble-average MSD scaling for the $\alpha_i = \sigma_i/\gamma$ that result from each of the previous σ_i and a fixed γ. (*B*) Predictions of the diffusion coefficient obtained by applying STEP to simulated ATTM trajectories (dots) and the result of applying the changepoint analysis (black line). (*C*) Distribution of D obtained through the analysis described in (*B*), showing the expected power-law behavior at small D. (*D*) Relation between D and the dwell time τ obtained through the analysis described in (*B*), showing the expected power-law behavior. (*E*) Examples of experimental trajectories of DC-SIGN with the corresponding predictions obtained for D (dots) and the changepoint analysis (black line). (*F*) Histogram of the distribution of D obtained for the experimental trajectories. Inset: power-law fit at small D. (*G*) 2D histogram of D and α obtained for the experimental trajectories. For details about the data used in each panel, see Appendix B3 and Table I therein. To see this figure in color, go online.

In brief, ATTM depicts Brownian diffusion randomly switching diffusion coefficient D for a given time τ (Fig. 4 *A*, top). This aims to mimic the spatio-temporal heterogeneities present in biological environments. Although particles are effectively performing Brownian diffusion at short scales, under particular distributions of D and τ, the diffusion is anomalous and weakly nonergodic at larger scales (62). For instance, one may consider that the values of D are sampled from a distribution with a power-law behavior $D^{\sigma-1}$ for small D and a fast decay for $D \to \infty$ (Fig. 4 *A*, middle). Moreover, assuming a correlation between D and the dwell time τ of the form $\tau(D) \sim D^{-\gamma}$ (inset of Fig. 4 *A*, middle) to predict an anomalous diffusion exponent $\alpha = \sigma/\gamma$ (Fig. 4 *A*, bottom). Therefore, the correct characterization of both the distribution of D and τ is crucial to corroborate the compatibility with the underlying model. In the original work (23), changes of diffusivity were detected through a changepoint analysis (63) but the sensitivity and the time resolution of the method did not allow a thorough investigation of this behavior.

To demonstrate that STEP enables a better characterization of these data, we first use simulated ATTM trajectories. We set $\sigma = 0.3$ and $\gamma = 0.4$ ($\alpha = 0.75$), resulting in trajectories with $D \in (10^{-6.7}, 10^0)$ with 18 different segments, on average, for trajectories of 200 time steps. We segment the trajectories applying the KCPD algorithm introduced in the previous sections over the STEP predictions of D, as we show in Fig. 4 *B*. Thus, we assign to each segment a single D, taking the average segment prediction, and a τ. We successfully recover the power-law behavior of D (Fig. 4 *C*) and the power-law relationship between τ and D (Fig. 4 *D*). The faint harmonic in Fig. 4 *D* corresponds to $2D^{-\gamma}$, which results from the missed detection of a changepoint between consecutive segments with very similar D (hence similar τ). Interestingly, when performing predictions of α, STEP predicts $\alpha \sim 1$, as expected from the properties of the diffusion model (see Appendix D).

Then, we apply this approach to the DC-SIGN trajectories of Ref. (23). The results confirm the occurrence of diffusivity changes between segments of nearly constant diffusion coefficient and with variable duration, as we show in Fig. 4 *E*. Interestingly, our approach reveals twice as many changepoints as the previous analysis.

The distribution of D obtained for trajectory segments spans several orders of magnitude, as we show in the histogram of Fig. 4 *F*. For small D, it displays a behavior compatible with a power law with exponent $\sigma \approx 0.37$ over nearly three decades (inset of Fig. 4 *F*), compatible with the ATTM. Notably, this behavior could not be directly verified in the original article. In principle, our method would allow us to verify the correlation between D and dwell time, as we have shown in the simulations. However, this task is limited by the variable trajectory length (64) and by the lack of statistics, in particular for segments at small D.

As a further test, we predict the anomalous diffusion exponent with STEP. We assign a single α by taking the average prediction of each segment. The results reported in Fig. 4 *G* show an interesting correlation between D and α that suggests a more complex diffusion pattern, involving the occurrence of anomalous diffusion also at the level of individual segments.

Characterizing multi-state diffusion processes

We use STEP to analyze experimental trajectories of the integrin $\alpha5\beta1$ diffusing in the membrane of HeLa cells

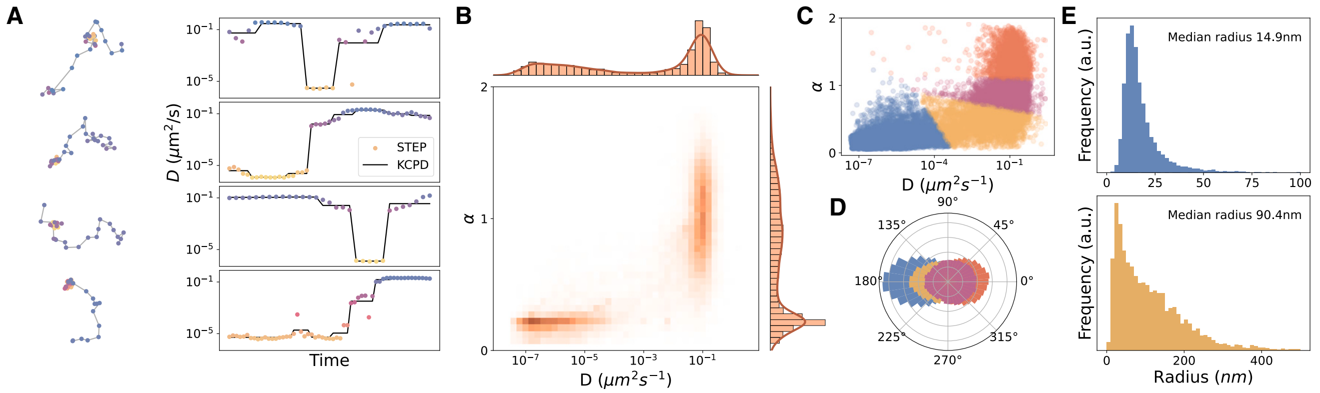

FIGURE 5 Multi-state diffusion of the integrin $\alpha5\beta1$. (*A*) Examples of experimental trajectories of the integrin $\alpha5\beta1$ with the corresponding predictions obtained for D (dots) and the changepoint analysis (black line). (*B*) 2D histogram of D and α with the respective marginal distributions. (*C*) Scatter plot of the predictions obtained for D and α at the segment level, color coded according to a clustering analysis performed with a k-means algorithm. (*D*) Distribution of the turning angle for the four clusters of segments obtained as in (*C*). (*E*) Distribution of the confinement radius for the clusters showing restrained diffusion. For details about the data in each panel, see Appendix B3 and Table I therein. To see this figure in color, go online.

(see Appendix E for experimental details). Integrins are transmembrane receptors for the extracellular matrix (ECM) in focal adhesions, which mechanically link the ECM and actin filaments in the cytoplasm and activate signaling pathways involved in cell migration, proliferation, or apoptosis (39). The dynamics of the integrin $\alpha5\beta1$ is influenced by interactions with fibronectin and actin-binding proteins (64,65). Its motion has been reported to switch from fast free diffusion to slow free diffusion and immobilization, as well as exhibiting rearward actin-driven movement.

We use STEP to predict both the diffusion coefficient and the anomalous diffusion exponent for the integrin $\alpha5\beta1$ trajectories. Then, we segment the trajectories by applying the KCPD method to both predictions at once. In this way, we assign every segment a unique D and α by taking the average prediction over the segment. Examples of the results are shown in Fig. 5 *A* and the joint distribution of D and α in Fig. 5 *B*. The visual inspection of Fig. 5 *B* reveals two main clusters centered around ($D = 10^{-6}\mu m^2/s$, $\alpha = 0.25$) and ($D = 0.1\ \mu m^2/s$, $\alpha = 1$). The 2D histogram of the same parameters calculated at the pointwise level (pre-segmentation) does not show any major differences with respect to Fig. 5 *B*.

Nonetheless, combining the k-means clustering algorithm with the elbow method (66), we find the data optimally separates in four clusters of segments (Fig. 5 *C*) characterized by different motion features. The first two clusters show a rather restrained motion, with integrins spending 40% of the time in a state characterized by $D = 1.2\times 10^{-5}\mu m^2/s$ and $\alpha = 0.23$, and 14% of the time with $D = 0.06\ \mu m^2/s$ and $\alpha = 0.46$. For both clusters, the distribution of angles between successive steps shows a peak centered at 180°, indicating backward movements due to reflection at potential boundaries, as we show in Fig. 5 *D*. The confinement radius of the first cluster has a median of 14.9 nm (SD = 13.3 nm), which is comparable to the localization precision of these experiments. This allows us to associate it with protein immobilization. The second cluster shows confined motion within areas with a broad distribution of sizes, as we see in Fig. 5 *E*, and a median radius of 90.4 nm (SD = 98.2 nm). The third cluster represents 29% of the total recording and shows minor deviations from Brownian motion with $\alpha = 0.88$ and a nearly uniform angle distribution, and it has an average $D = 0.10\ \mu m^2/s$, close to the value typically reported for this protein. Interestingly, the analysis pinpoints a fourth population, corresponding to 20% of the total recording, undergoing superdiffusion with $\alpha = 1.3$ and $D = 0.14\ \mu m^2/s$, and with a persistent direction of motion between consecutive steps (Fig. 5 *D*).

DISCUSSION

In this work, we present STEP, a machine-learning method to predict diffusion properties from individual trajectories at every time step. The method relies on a combination of state-of-the-art machine-learning architectures that take into account correlations at different timescales. The presented approach is especially appealing to analyze trajectories from particles undergoing heterogeneous motion, where changes in diffusion properties occur over time. Moreover, it does not require prior knowledge of the underlying physical process or the temporal resolution at which changes in diffusion occur.

To illustrate the power of STEP, we benchmark it on simulated trajectories under various conditions. We show its ability to predict piecewise constant diffusion properties, such as the diffusion coefficient or the anomalous diffusion exponent, in noisy and short trajectories. Furthermore, we demonstrate that STEP boosts the accuracy of a changepoint detection algorithm to detect the time at which diffusion

changes take place. Importantly, we also prove the suitability of our method to study continuous changes of diffusion.

To further showcase the potential applications of the method, we study trajectories obtained by tracking live-cell single-molecule imaging experiments of proteins of the plasma membrane. First, we characterize the motion of the pathogen-recognition receptor DC-SIGN, which was shown to exhibit random changes in the diffusion coefficient. Our analysis confirms such a hypothesis and improves the accuracy with which we detect these changes. Moreover, our results suggest the occurrence of more complex phenomena that need further investigation. Second, we study the diffusion of the integrin $\alpha_5\beta_1$. In agreement with previous works, our analysis confirms the existence of different diffusion modes and allows their precise classification according to the diffusion coefficient, the anomalous diffusion exponent, and the levels of spatial constraint.

We believe that STEP represents a first step toward a new class of machine-learning algorithms to study dynamic systems through a sequence-to-sequence approach. The instantaneous prediction of the property of interest enables the characterization of the trajectories at experimental time resolution without averaging and filtering and minimizes the prior knowledge needed to perform the analysis. As such, the results obtained with STEP can provide information about diffusion properties with unprecedented resolution and thus shed light on the underlying physical processes of a variety of systems. One of the primary advantages of STEP is its broad applicability. However, as demonstrated in the "results" section, specialized methods may produce more accurate results when applied specifically to their intended tasks. Consequently, a significant benefit of STEP is its potential integration with these methods, facilitating their utilization across a wider spectrum of scenarios, such as enhancing trajectory segmentation.

DATA AND CODE AVAILABILITY

All the resources relative to the machine-learning model are accessible in the public repository from Ref. (57). The experimental data are available upon request.

SUPPORTING MATERIAL

Supporting material can be found online at https://doi.org/10.1016/j.bpj.2023.10.015.

AUTHOR CONTRIBUTIONS

Conceptualization, B.R. and G.M.G.; methodology, B.R., G.M.G., and C.M.; software, B.R.; investigation, S.M., J.B., and C.M.; formal analysis, B.R., G.M.G., and C.M.; supervision, G.M.G., M.L., J.B., and C.M.; writing, B.R., G.M.G., M.L., and C.M.

ACKNOWLEDGMENTS

The authors acknowledge Montserrat Masoliver-Prieto and Marta Cullell-Dalmau for their valuable help with the experimental procedures.

Funding: GMG acknowledges support from the European Union and the Austrian Science Fund (F.W.F.) through the SFB BeyondC F7102. C.M. acknowledges support through grant RYC-2015-17896 funded by MCIN/AEI/10.13039/501100011033 and "ESF Investing in your future", grants BFU2017-85693-R and PID2021-125386NB-I00 funded by MCIN/AEI/10.13039/501100011033/ and "ERDF A way of making Europe", and grant AGAUR 2017SGR940 funded by the Generalitat de Catalunya. B.R. and M.L. acknowledge support from ERC AdG NOQIA; Ministerio de Ciencia y Innovación Agencia Estatal de Investigaciones (PGC2018-097027-B-I00/10.13039/501100011033, CEX2019-000910-S/10.13039/501100011033, Plan Nacional FIDEUA PID2019-106901GB-I00, Plan Nacional STAMEENA PID2022-139099NB-I00 project funded by the MICIN/AEI/10.13039/501100011033/FEDER, EU, FPI); MCIN/AEI/10.13039/501100011033 and by the "European Union NextGenerationEU/PRTR" (PRTR-C17.I1): QUANTERA MAQS (PCI2019-111828-2); QUANTERA DYNAMITE PCI2022-132919 (QuantERA II Programme co-funded by European Union's Horizon 2020 programme under Grant Agreement No 101017733), Ministry of Economic Affairs and Digital Transformation of the Spanish Government through the QUANTUM ENIA project call – Quantum Spain project, and by the European Union through the Recovery, Transformation and Resilience Plan – NextGenerationEU within the framework of the Digital Spain 2026 Agenda. Fundació Cellex; Fundació Mir-Puig; Generalitat de Catalunya (European Social Fund FEDER and CERCA program, AGAUR Grant No. 2021 SGR 01452, QuantumCAT \ U16-011424, co-funded by ERDF Operational Program of Catalonia 2014-2020); Barcelona Supercomputing Center MareNostrum (FI-2023-1-0013); EU Quantum Flagship (PASQuanS2.1, 101113690); EU Horizon 2020 FET-OPEN OPTOlogic (Grant No 899794); EU Horizon Europe Program (Grant Agreement 101080086 — NeQST), ICFO Internal "QuantumGaudi" project; European Union's Horizon 2020 program under the Marie Sklodowska-Curie grant agreement No 847648; "La Caixa" Junior Leaders fellowships, La Caixa" Foundation (ID 100010434): LCF/BQ/PR23/11980043. Views and opinions expressed in this work are, however, those of the author(s) only and do not necessarily reflect those of the European Union, European Climate, Infrastructure and Environment Executive Agency (CINEA), or any other granting authority. Neither the European Union nor any granting authority can be held responsible for them.

DECLARATION OF INTERESTS

The authors declare that they have no competing interests.

REFERENCES

1. Möckl, L., D. C. Lamb, and C. Bräuchle. 2014. Super-resolved fluorescence microscopy: nobel prize in chemistry 2014 for eric betzig, stefan hell, and william e. moerner. *Angew. Chem. Int. Ed.* 53:13972–13977.
2. Felix, H., and T. Franosch. 2013. Anomalous transport in the crowded world of biological cells. *Rep. Prog. Phys.* 76, 046602.
3. Bechinger, C., R. Di Leonardo, …, G. Volpe. 2016. Active particles in complex and crowded environments. *Rev. Mod. Phys.* 88, 045006.
4. Vilk, O., D. Campos, …, M. Assaf. 2022. Phase transition in a non-markovian animal exploration model with preferential returns. *Phys. Rev. Lett.* 128, 148301.
5. Manzo, C., and M. F. Garcia-Parajo. 2015. A review of progress in single particle tracking: from methods to biophysical insights. *Rep. Prog. Phys.* 78, 124601.

6. Michalet, X., and A. J. Berglund. 2012. Optimal diffusion coefficient estimation in single-particle tracking. *Phys. Rev.* 85, 061916.

7. Vestergaard, C. L., P. C. Blainey, and H. Flyvbjerg. Feb 2014. Optimal estimation of diffusion coefficients from single-particle trajectories. *Phys. Rev. E.* 89, 022726.

8. Metzler, R., J.-H. Jeon, …, E. Barkai. 2014. Anomalous diffusion models and their properties: non-stationarity, non-ergodicity, and ageing at the centenary of single particle tracking. *Phys. Chem. Chem. Phys.* 16:24128–24164.

9. Martin, D. S., M. B. Forstner, and J. A. Käs. 2002. Apparent subdiffusion inherent to single particle tracking. *Biophys. J.* 83:2109–2117.

10. Kepten, E., I. Bronshtein, and Y. Garini. 2013. Improved estimation of anomalous diffusion exponents in single-particle tracking experiments. *Phys. Rev.* 87, 052713.

11. Kepten, E., A. Weron, …, Y. Garini. 2015. Guidelines for the fitting of anomalous diffusion mean square displacement graphs from single particle tracking experiments. *PLoS One.* 10, e0117722.

12. Krapf, D., N. Lukat, …, X. Xu. 2019. Spectral content of a single non-brownian trajectory. *Phys. Rev. X.* 9, 011019.

13. Sposini, V., D. Krapf, …, G. Oshanin. 2022. Towards a robust criterion of anomalous diffusion. *Commun. Phys.* 5:305.

14. Thapa, S., M. A. Lomholt, …, R. Metzler. 2018. Bayesian analysis of single-particle tracking data using the nested-sampling algorithm: maximum-likelihood model selection applied to stochastic-diffusivity data. *Phys. Chem. Chem. Phys.* 20:29018–29037.

15. Muñoz-Gil, G., G. Volpe, C. Manzo…, 2021. Objective comparison of methods to decode anomalous diffusion. *Nat. Commun.* 12:6253.

16. Yin, S., N. Song, and H. Yang. 2018. Detection of velocity and diffusion coefficient change points in single-particle trajectories. *Biophys. J.* 115:217–229.

17. Saha, S., I.-H. Lee, …, S. Mayor. 2015. Diffusion of gpi-anchored proteins is influenced by the activity of dynamic cortical actin. *Mol. Biol. Cell.* 26:4033–4045.

18. Bag, N., S. Huang, and T. Wohland. 2015. Plasma membrane organization of epidermal growth factor receptor in resting and ligand-bound states. *Biophys. J.* 109:1925–1936.

19. Low-Nam, S. T., K. A. Lidke, …, D. S. Lidke. 2011. Erbb1 dimerization is promoted by domain co-confinement and stabilized by ligand binding. *Nat. Struct. Mol. Biol.* 18:1244–1249.

20. Sabri, A., X. Xu, …, M. Weiss. 2020. Elucidating the origin of heterogeneous anomalous diffusion in the cytoplasm of mammalian cells. *Phys. Rev. Lett.* 125, 058101.

21. Jeon, J.-H., M. Javanainen, …, I. Vattulainen. 2016. Protein crowding in lipid bilayers gives rise to non-gaussian anomalous lateral diffusion of phospholipids and proteins. *Phys. Rev. X.* 6, 021006.

22. Lampo, T. J., S. Stylianidou, …, A. J. Spakowitz. 2017. Cytoplasmic rna-protein particles exhibit non-gaussian subdiffusive behavior. *Biophys. J.* 112:532–542.

23. Manzo, C., J. A. Torreno-Pina, …, M. F. Garcia Parajo. 2015. Weak ergodicity breaking of receptor motion in living cells stemming from random diffusivity. *Phys. Rev. X.* 5, 011021.

24. Jeon, J.-H., A. V. Chechkin, and R. Metzler. 2014. Scaled brownian motion: a paradoxical process with a time dependent diffusivity for the description of anomalous diffusion. *Phys. Chem. Chem. Phys.* 16:15811–15817.

25. Vega, A. R., S. A. Freeman, …, K. Jaqaman. 2018. Multistep track segmentation and motion classification for transient mobility analysis. *Biophys. J.* 114:1018–1025.

26. Lanoiselée, Y., and D. S. Grebenkov. 2017. Unraveling intermittent features in single-particle trajectories by a local convex hull method. *Phys. Rev. E.* 96, 022144.

27. Bronson, J. E., J. Fei, …, C. H. Wiggins. 2009. Learning rates and states from biophysical time series: a bayesian approach to model selection and single-molecule fret data. *Biophys. J.* 97:3196–3205.

28. Persson, F., M. Lindén, …, J. Elf. 2013. Extracting intracellular diffusive states and transition rates from single-molecule tracking data. *Nat. Methods.* 10:265–269.

29. Monnier, N., Z. Barry, …, M. Bathe. 2015. Inferring transient particle transport dynamics in live cells. *Nat. Methods.* 12:838–840.

30. Verdier, H., F. Laurent, …, J.-B. Masson. 2022. Variational inference of fractional brownian motion with linear computational complexity. *Phys. Rev. E.* 106, 055311.

31. Pineda, J., B. Midtvedt, …, C. Manzo. 2023. Geometric deep learning reveals the spatiotemporal features of microscopic motion. *Nat. Mach. Intell.* 5:71–82.

32. Arts, M., I. Smal, …, E. Meijering. 2019. Particle mobility analysis using deep learning and the moment scaling spectrum. *Sci. Rep.* 9:17160–17210.

33. Sutskever, I., Oriol Vinyals, and V. L. Quoc. 2014. Sequence to sequence learning with neural networks. *In* Advances in Neural Information Processing Systems, *volume 27*. Z. Ghahramani, M. Welling, and …, K. Q. Weinbergereds. Curran Associates, Inc..

34. Brown, T., B. Mann, …, A. Askell. 2020. Language models are few-shot learners. *Adv. Neural Inf. Process. Syst.* 33:1877–1901.

35. Scott, R., K. Zolna, …, N. de Freitas. 2022. A Generalist Agent. *In* Transactions on Machine Learning Research Featured Certification, Outstanding Certification.

36. Taylor, R., M. Kardas, …, Robert Stojnic. 2022. Galactica: A large language model for science. Preprint at arXiv. https://doi.org/10.48550/arXiv.2211.09085.

37. LeCun, Y., B. Boser, …, L. D. Jackel. 1989. Backpropagation applied to handwritten zip code recognition. *Neural Comput.* 1:541–551.

38. Vaswani, A., N. Shazeer, …, I. Polosukhin. 2017. Attention is all you need. *In* Advances in Neural Information Processing Systems.

39. Kanchanawong, P., and A. C. David. 2022. Organization, dynamics and mechanoregulation of integrin-mediated cell–ecm adhesions. *Nat. Rev. Mol. Cell Biol.* 1–20

40. Muñoz-Gil, G., M. A. Garcia-March, …, M. Lewenstein. 2020. Single trajectory characterization via machine learning. *New J. Phys.* 22, 013010.

41. Granik, N., L. E. Weiss, …, Y. Shechtman. 2019. Single-particle diffusion characterization by deep learning. *Biophys. J.* 117:185–192.

42. Bo, S., F. Schmidt, …, G. Volpe. Jul 2019. Measurement of anomalous diffusion using recurrent neural networks. *Phys. Rev. E.* 100, 010102.

43. Kowalek, P., H. Loch-Olszewska, and J. Szwabiński. 2019. Classification of diffusion modes in single-particle tracking data: Feature-based versus deep-learning approach. *Phys. Rev. E.* 100, 032410.

44. Seckler, H., and R. Metzler. 2022. Bayesian deep learning for error estimation in the analysis of anomalous diffusion. *Nat. Commun.* 13:6717.

45. Jamali, V., C. Hargus, …, A. P. Alivisatos. 2021. Anomalous nanoparticle surface diffusion in lctem is revealed by deep learning-assisted analysis. *Proc. Natl. Acad. Sci. USA.* 118, e2017616118.

46. Muñoz-Gil, G., C. Romero-Aristizabal, …, J. A. Torreno-Pina. 2022. Stochastic particle unbinding modulates growth dynamics and size of transcription factor condensates in living cells. *Proc. Natl. Acad. Sci. USA.* 119, e2200667119.

47. Gentili, A., and G. Volpe. 2021. Characterization of anomalous diffusion classical statistics powered by deep learning (condor). *J. Phys. Math. Theor.* 54, 314003.

48. Argun, A., G. Volpe, and S. Bo. 2021. Classification, inference and segmentation of anomalous diffusion with recurrent neural networks. *J. Phys. Math. Theor.* 54, 294003.

49. Héctor Buena Maizón, Barrantes, F. J. 2021. A deep learning-based approach to model anomalous diffusion of membrane proteins: the case of the nicotinic acetylcholine receptor. *Briefings Bioinf.* 23:10.

50. Verdier, H., M. Duval, …, J.-B. Masson. 2021. Learning physical properties of anomalous random walks using graph neural networks. *J. Phys. Math. Theor.* 54, 234001.

51. Manzo, C. 2021. Extreme learning machine for the characterization of anomalous diffusion from single trajectories (AnDi-ELM). *J. Phys. Math. Theor.* 54, 334002.
52. Li, D., Q. Yao, and Z. Huang. 2021. Wavenet-based deep neural networks for the characterization of anomalous diffusion (WADNet). *J. Phys. Math. Theor.* 54, 404003.
53. Garibo-i-Orts, Ò., A. Baeza-Bosca, …, J. A. Conejero. 2021. Efficient recurrent neural network methods for anomalously diffusing single particle short and noisy trajectories. *J. Phys. Math. Theor.* 54, 504002.
54. Firbas, N., Ò. Garibo-i-Orts, …, J. A. Conejero. 2023. Characterization of anomalous diffusion through convolutional transformers. *J. Phys. Math. Theor.* 56, 014001.
55. Kabbech, H., and I. Smal. 2022. Identification of diffusive states in tracking applications using unsupervised deep learning methods. *In* 2022 IEEE 19th International Symposium on Biomedical Imaging (ISBI) IEEE.
56. Tong, H., Z. Zhang, …, L. Mu. 2019. Bag of tricks for image classification with convolutional neural networks. *In* Proceedings of the IEEE/CVF Conference on Computer Vision and Pattern Recognition, pp. 558–567.
57. Requena, B., and G. Muñoz-Gil. 2022. Step python Library. https://github.com/borjarequena/step.
58. Celisse, A., G. Marot, …, G. J. Rigaill. 2018. New efficient algorithms for multiple change-point detection with reproducing kernels. *Comput. Stat. Data Anal.* 128:200–220.
59. Arlot, S., A. Celisse, and Z. Harchaoui. 2019. A kernel multiple change-point algorithm via model selection. *J. Mach. Learn. Res.* 20:1–56.
60. Mandelbrot, B. B., and J. W. Van Ness. 1968. Fractional brownian motions, fractional noises and applications. *SIAM Rev.* 10:422–437.
61. Manzo, C., J. A. Torreno-Pina, …, A. Cambi. 2012. The neck region of the c-type lectin dc-sign regulates its surface spatiotemporal organization and virus-binding capacity on antigen-presenting cells. *J. Biol. Chem.* 287:38946–38955.
62. Massignan, P., C. Manzo, …, G. Lapeyre. 2014. Nonergodic subdiffusion from brownian motion in an inhomogeneous medium. *Phys. Rev. Lett.* 112, 150603.
63. Montiel, D., H. Cang, and H. Yang. 2006. Quantitative characterization of changes in dynamical behavior for single-particle tracking studies. *J. Phys. Chem. B.* 110:19763–19770.
64. Tsunoyama, T. A., Y. Watanabe, …, A. Kusumi. 2018. Super-long single-molecule tracking reveals dynamic-anchorage-induced integrin function. *Nat. Chem. Biol.* 14:497–506.
65. Rossier, O., V. Octeau, …, G. Giannone. 2012. Integrins β1 and β3 exhibit distinct dynamic nanoscale organizations inside focal adhesions. *Nat. Cell Biol.* 14:1057–1067.
66. Satopaa, V., J. Albrecht, B. Raghavan…, 2011. Finding a “kneedle” in a haystack: Detecting knee points in system behavior. *In* 2011 31st International Conference on Distributed Computing Systems Workshops IEEE, pp. 166–171.

Biophysical Journal, Volume *122*

Supplemental information

Inferring pointwise diffusion properties of single trajectories with deep learning

Borja Requena, Sergi Masó-Orriols, Joan Bertran, Maciej Lewenstein, Carlo Manzo, and Gorka Muñoz-Gil

Appendices of "*Inferring pointwise diffusion properties of single trajectories with deep learning*"

Borja Requena,[1] Sergi Masó,[2] Joan Bertran,[2] Maciej Lewenstein,[1,3] Carlo Manzo,[2,*] and Gorka Muñoz-Gil[4,†]

[1]*ICFO – Institut de Ciències Fotòniques, The Barcelona Institute of Science and Technology, Av. Carl Friedrich Gauss 3, 08860 Castelldefels (Barcelona), Spain*

[2]*Facultat de Ciències, Tecnologia i Enginyeries, Universitat de Vic – Universitat Central de Catalunya (UVic-UCC), C. de la Laura,13, 08500 Vic, Spain*

[3]*ICREA, Pg. Lluís Companys 23, 08010 Barcelona, Spain*

[4]*Institute for Theoretical Physics, University of Innsbruck, Technikerstr. 21a, A-6020 Innsbruck, Austria*

Appendix A: Diffusion properties

In this section, we briefly highlight some of the main characteristics of normal and anomalous diffusion. We refer the reader to Refs. [1, 2] for a nice and thorough introduction to the field.

Diffusion trajectories are often described by means of their mean squared displacement (MSD) which, in the case of Brownian motion, shows a linear scaling with time, i.e. MSD $\propto Dt$, where D is the diffusion coefficient. However, there can be deviations from such linear scaling, resulting in a power-law relation between the MSD and time, i.e. MSD $\propto K_\alpha t^\alpha$, where α is defined as the anomalous diffusion exponent and K_α is an effective diffusion coefficient. The former allows us to distinguish between normal (or Brownian) diffusion ($\alpha = 1$) and anomalous diffusion ($\alpha \neq 1$).

The appearance of anomalous diffusion can be associated with very different phenomena, from the arising of correlations in the motion of the diffusing particle to the presence of spatiotemporal heterogeneity. To account for most of these phenomena, we follow Ref. [3] and consider five anomalous diffusion models with specific ranges for the anomalous diffusion exponent: annealed transient time model (ATTM) [4] with $\alpha \in [0.05, 1]$, continuous-time random walk (CTRW) [5] with $\alpha \in [0.05, 1]$, fractional Brownian motion (FBM) [6] with $\alpha \in [0.05, 1.95]$, Lévy walk (LW) [7] with $\alpha \in [1.05, 2]$, and scaled Brownian motion (SBM) [8] with $\alpha \in [0.05, 2]$.

Appendix B: Machine learning pipeline

Here, we provide a detailed explanation of the machine learning approach followed to obtain the results described throughout this work.

As we briefly mention in the main text, we train two different models: one for the diffusion coefficient task, and one for the anomalous diffusion exponent. We report the results regarding the prediction of the diffusion coefficient and anomalous diffusion exponent in Results. We implement both models following the same principles with very minor differences. In this section, we describe the architecture and the training process that we follow and, when needed, highlight the differences between models.

We provide the source code with extended explanations on how to reproduce the results in [9]. We make extensive use of the *PyTorch* [10] and *fastai* [11] libraries to implement the architecture and the training procedure. The kernel changepoint detection method (KCPD) was implemented using the *ruptures* Python library [12].

1. Architecture details

We propose to use a model that takes a trajectory $\mathbf{x}$ as input and outputs the target diffusion properties at each time step. The input trajectory is a d-dimensional vector of arbitrary length T, whose elements, x_t, correspond to the particle position at every time step t. Then, the output is a one-dimensional vector of length T, whose elements correspond to the diffusion property of interest at every time step, e.g., D_t in the case of the diffusion coefficient. See Fig. 1 for further details about the dimensions. Throughout this work, we mainly consider trajectories of dimension $d = 2$.

The model we propose consists of three main modules: an initial convolutional part that processes the input trajectory; a self-attention-based part that feeds on the features extracted by the previous one; a shallow pointwise fully connected feedforward module that provides the desired output dimensions. The entire architecture is length independent, which allows us to process trajectories of arbitrary lengths.

Convolutional module – The first main convolutional module allows us to expand the trajectory dimension with several convolutional filters. This provides the following layers with a richer embedding based on short-range correlations.

We build it following the XResNet [13] architecture. As we show in Fig. 1, it consists of an initial convolutional layer, commonly referred to as the *stem*, followed by a series of *residual blocks* that feature a convolutional layer with a skip connection. We use one-dimensional convolutions with a kernel size of three and stride one to preserve the trajectory size. However, we use a

* carlo.manzo@uvic.cat
† munoz.gil.gorka@gmail.com

kernel size of one in the skip connections, which can act as the identity or a scaling factor whose main purpose is to match the tensor shapes on both paths of the residual blocks, as we explain below. This module can take a batch of input trajectories of size [`batch_size` $\times T \times d$] and output a batch of features of size [`batch_size` $\times T \times$ `embedding_size`].

Throughout the architecture, we add a batch normalization layer directly after every convolutional layer, and we use the rectified linear unit (ReLU) activation function by default, except in the last output layer.

To produce the results, we use a single convolutional layer and a ReLU activation in the stem. We use 64 filters to predict the diffusion coefficient and 32 filters for the anomalous diffusion exponent. Then, we have added three residual blocks with 128, 256 and 512 filters, respectively. Hence, `embedding_size` $= 512$. In these blocks, the convolutional paths have two convolutional layers: the first one increases the embedding size and the second one preserves the dimensions. In the skip connection, we only have one convolutional layer that increases the embedding size to match the dimensions of the convolutional path. We implement the ReLU activation at the end of the block, after we add the outcome of both paths.

Self-attention module – We process the features extracted by the convolutional module with a self-attention mechanism that allows the model to capture long-range correlations.

More precisely, we implement a *transformer encoder*, as it was introduced in Ref. [14]. As we illustrate in Fig. 1, the encoder block has two main parts, both featuring a skip connection followed by a layer normalization after the sum of both paths. In the first one, we have a multi-head attention layer that feeds on the input and, in the second one, we have a couple of pointwise feedforward layers, which are equally applied to each element in the incoming tensor. Furthermore, we can add a positional encoding before the first encoder block, which provides information about the relative position of each element in the trajectory. This module can process a batch of embeddings preserving its dimensions. Hence, the input and the output both have size [`batch_size` $\times T \times$ `embedding_size`].

To produce the results, we use four transformer encoder blocks with eight heads in the multi-head attention layers. The pointwise feedforward part adds two fully-connected layers with `embedding_size` neurons each, i.e, 512 in this case. Interestingly, we have found that, after the convolutions, the positional encoding has very little impact on the results. Therefore, in the interest of simplicity, we have not used it to obtain the results reported in this work.

Feedforward module – The last main part is a shallow feedforward fully-connected network that acts element-wise on the features extracted by the previous module. We tailor this part to the specific task at hand to achieve the desired output with the proper dimensions.

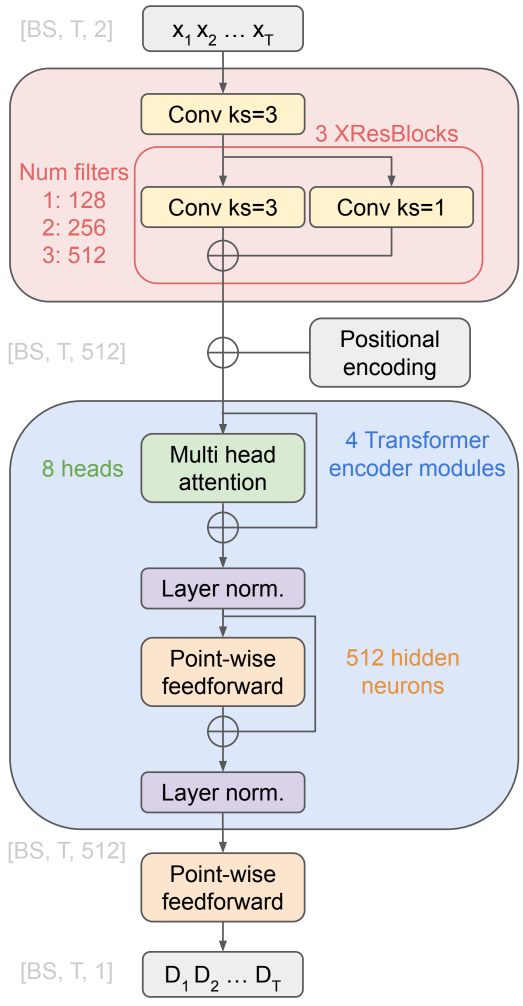

Figure 1. Machine learning architecture representation

For instance, in a regression task, the output dimension is one and we use a scaled sigmoid activation function at the end to define the output range with some margin, e.g., $\log D \in (-3.1, 3.1)$, $\alpha \in (0, 2.05)$. This margin allows the sigmoid to reach the desired values before it saturates. In a hypothetical case of classification task (as e.g. classifying between diffusion models as done in Ref. [3]), the final dimension is the number of classes and we use a softmax activation function. Then, we obtain the predictions by choosing the class with the maximum activation value. Hence, we can process a feature batch of size [`batch_size` $\times T \times$ `embedding_size`] and output their predictions with size [`batch_size` $\times T \times$ `num_class`]. In case that `num_class` > 1, as in a classification task, we perform an additional post-processing step to obtain an output of size [`batch_size` $\times T \times 1$] with the corresponding predictions at each time step.

2. Training procedure

We follow a standard gradient-based training procedure for both of our models. The only differences between them arise from the training data and how we process it.

The main training loop consists on:

1. Predict the values over a batch of training data.
2. Compute the loss function with respect to the true values.
3. Update the model parameters based on the loss gradient.

We use batches containing 128 trajectories and the L1 loss function, which corresponds to the mean absolute error. Formally,

$$\mathcal{L}_{\mathrm{MAE}}(\mathbf{x}) = \frac{1}{n\sum_i T_i}\sum_{i=1}^{n}\sum_{t=1}^{T_i}\left|y_{i,t} - f(\mathbf{x}_i)_t\right| \,, \tag{B1}$$

where $f(\mathbf{x}_i)_t$ denotes the prediction of the i-th trajectory at the time step t in a batch of n trajectories. $y_{i,t}$ denotes its true label for the same time, and T_i denotes its length.

To perform the parameter update, we use an Adam [15] optimizer. We use the fastai [11] library to choose the learning rate with the learning rate finder tool, typically of the order of 10^{-4}. Then, we implement a schedule over the training batches both in the learning rate and its momentum, following the one-cycle policy introduced in Refs. [16, 17]. We train our models until the performance in the validation set stabilizes, typically between ten to twenty epochs.

To further prevent overfitting and enhance the model generalization capabilities, we use dropout [18] and weight decay [19, 20]. Additionally, we add Gaussian localization noise at different intensities to the trajectories as a form of data augmentation.

3. Data

In order to properly evaluate our models, we generate several independent data sets. We use one to train and validate our models, and we use the others to test them on unseen scenarios. All the results that we report throughout this work are obtained using the test sets, which we design to evaluate different aspects of our models.

In Table I, we provide the details about the data sets that we use to train, validate and test our models. These data sets contain simulated trajectories with their corresponding labels at each time step. We have two main approaches to simulate the trajectories depending on whether we deal with normal or anomalous diffusion. Below, we explain how we generate the data for both cases.

While there are some differences between how we simulate and label our trajectories for normal and anomalous diffusion, there are several common factors that hold for all of them. For instance, all segments have constant diffusion properties and they are, at least, 10-time steps long.

Brownian motion – We simulate Brownian motion trajectories by taking uncorrelated Gaussian noise as the trajectory displacements. We control the diffusion coefficient at each time step with the standard deviation of the Gaussian noise, which corresponds to $\sqrt{2D}$. This way, we can easily generate segments of arbitrary lengths with a constant diffusion coefficient, D, along the trajectories. Finally, we perform the cumulative sum of the displacements to obtain the trajectory coordinates and we subtract the initial position such that they start at the origin.

We consider diffusion coefficients across six orders of magnitude $D \in [10^{-3}, 10^{3}]$. However, we take its logarithm as labels for the regression task, such that $y_i \in [-3, 3]$ at every time step. This greatly simplifies the problem and allows us to keep a consistent performance across all orders of magnitude.

Additionally, we can simulate experimental localization noise by adding Gaussian noise with standard deviation σ_{noise}. We use this as a form of data augmentation during training and to study the model's resilience to noise. See Table I for further details.

Anomalous diffusion – To simulate anomalous diffusion trajectories, we consider the five diffusion models introduced in Appendix A with their respective anomalous diffusion exponent ranges. We generate full trajectories for each model following the same procedure detailed in the Supplementary Material from Ref. [3] and using the library provided by the authors [22]. Then, in order to obtain heterogeneous trajectories, we split them into segments and combine them together. We impose the condition that two consecutive segments must differ, at least, either in the diffusion model or the anomalous diffusion exponent. Finally, we add Gaussian localization noise, with standard deviation σ_{noise}. Then, we normalize the resulting displacements by their standard deviation and subtract the initial position to ensure that the trajectory starts at the origin.

Therefore, we have two labels at each time step: the anomalous diffusion exponent and the diffusion model with which the corresponding segment was generated. This allows us to use the same data for both a regression task in the anomalous diffusion exponent and a classification task in the diffusion model. However, in this work, we have mainly focused on the first one. Furthermore, we balance all the data sets such that there is an even representation of both the anomalous diffusion exponents and diffusion models throughout all the time steps.

Task	Models	D	α	σ_{noise}	Traj. length	Segments	Seg. length	Size
**Train BM**	Brownian motion	$[-3,3]$	1	$[-6,2]$	200	$[2,5]$	$[10,190]$	100,000
**Train AnDi**	all anomalous	1	$[0.05,2]$	0.1	200	$[2,5]$	$[10,190]$	100,064
Fig. 2A & B, Fig. 3D	Brownian motion	$[-3,3]$	1	0	200	$[2,5]$	$[10,190]$	48,000
Fig. 2C, Fig. 4 Fig. 3C & D	all anomalous	1	$[0.05,2]$	$[-5,2]$	200	$[2,5]$	$[10,190]$	49,994
Fig. 2D, Fig. 3A & B	all anomalous	1	$[0.05,2]$	$\{0,0.1\}$	200	$[2,5]$	$[10,190]$	50,000
Fig. 2E & F	Brownian motion	$[-3,3]$	1	0	200	2	$[10,190]$	50,000
Fig. 2G & H	FBM	1	$[0.05,1.95]$	0	200	2	$[10,190]$	40,000
Fig. 3	SBM	1	$\{0.1,0.5\}$	0	200	1	200	6,000
Fig. 4B, C & D	ATTM	(-6.7, 0)	0.75	0	200	$[1,51]$	$[1,200]$	10,000
Fig. 4E, F & G	(experiment)				$[200,2000]$			755
Fig. 5	(experiment)				$[20,500]$			4734
Fig. 2A	Brownian motion	$[-3,3]$	1	0	$[20,660]$	$[1,11]$	$\{20,40,60\}$	22,000
Fig. 2B	Brownian motion	$[-3,3]$	1	0	$[40,660]$	$[2,11]$	$\{20,40,60\}$	20,000
Fig. 2C	Brownian motion	$[-3,3]$	1	$[-6,0]$	200	$[2,5]$	$[10,190]$	48,000
Fig. 2D	Brownian motion	$[-3,3]$	1	0	200	[2, 5]	[10, 190]	200,384

Table I. **Data set details for all the results reported throughout this paper.** The ranges for D and σ_{noise} are in $\log_{10}$ scale, and we take α intervals of 0.05 within the denoted ranges. The values of D are logarithmically spaced and we take 1000 unique values unless stated otherwise. All the datasets with 2 to 5 segments have their lengths sampled according to an exponential distribution with a minimum length of 10 and a maximum of 190 steps, with an average of ~ 57 time steps. We use 20% of the training data (first two rows) for validation and hyperparameter tuning, whereas the rest is used for testing. We use the same Brownian motion test set from Fig. 2A & B to predict α for Fig. 3D. We take two independent sub-samples of a test set with 199,976 trajectories: one for Fig. 2C, Fig. 3C & D, Fig. 4 and the other for Fig. 2D, Fig. 3A & B. In Fig. 2D, we consider noiseless trajectories, although we add noise with $\sigma_{\text{noise}} = 0.1$ to flat CTRW segments that would result in numerical instabilities for the TA-MSD method. To generate the noisy trajectories for Fig. 2C, Fig. 3C & D and Fig. 4, we add 128 random levels of localization noise to each trajectory in the data set, effectively making about 6.4×10^6 trajectories. Fig. 2C is decomposed in Fig. 4 and, thus, uses the same data. In Fig. 3, we evenly split the two values of α among all trajectories. In Fig. 4B, C & D we use ATTM trajectories with $\sigma = 0.3$ and $\gamma = 0.4$ (see [4, 21], do not confuse with σ_{noise}). We simulate them randomly sampling D and the segment lengths accordingly, and the values here come from analysing the resulting trajectories, which have 18 different segments on average. For Fig. 4E, F & G we consider 755 experimental trajectories of the pathogen-recognition receptor DC-SIGN containing from 200 to 2000 frames sampled at 60 Hz. We obtain all the results in Fig. 5 from 4734 trajectories of the integrin $\alpha 5\beta 1$ containing from 20 to 500 frames sampled at 33 Hz. The data set for Fig. 2B is a sub-set of the one from Fig. 2A. In Fig. 2C, we use the same trajectories from Fig. 2A & B and add 128 random levels of localization noise to each of them, effectively making 6.144×10^6 noisy trajectories. In Fig. 2D, we average over all D values for each segment length to estimate the uncertainty. We take D in intervals of 0.2 within the given range (31 values), which combined with over 2×10^5 trajectories (over 7×10^5 segments combined) result in ~ 125 segments per D and length value.

Appendix C: Diffusion coefficient prediction

In the Results section, we study the capability of STEP to properly infer the diffusion coefficient at every time step and detect changes in diffusive behavior. Here, we complement the analysis presented in the main text by considering additional factors that impact the performance, such as the number of segments in the trajectories and the localization noise, typical of experimental setups. We show the results in Fig. 2.

We investigate the effect of the number of segments on the characterization of the trajectories. To test it, we fix the segment length and generate trajectories with one to eleven segments (zero to ten changepoints), resulting in trajectories with very different lengths (see Appendix B 3 for details). In Fig. 2A, we see a slight increase of the relative error with the number of segments, although it has a much lesser impact than the segment length, e.g., it is harder to characterize a single segment of twenty points than eight consecutive segments of 40 time steps. Importantly, even in the presence of 10 changepoints, STEP still heavily outperforms the TA-MSD approach applied to segments (no changes) of the same size, e.g., the whole curve for a segment length of 20 in Fig. 2A is well below the TA-MSD point for a segment length of 20 in Fig. 2C.

In Fig. 2E and F, we show how to combine STEP with a KCPD method to detect diffusion changes. In Fig. 2B, we show the performance as a function of the number of segments. We see that the shortest segments are the hardest to characterize. However, segment length becomes less important for sufficiently long ones, as the curves for lengths 40 and 60 behave fairly similarly. We see that STEP achieves a better

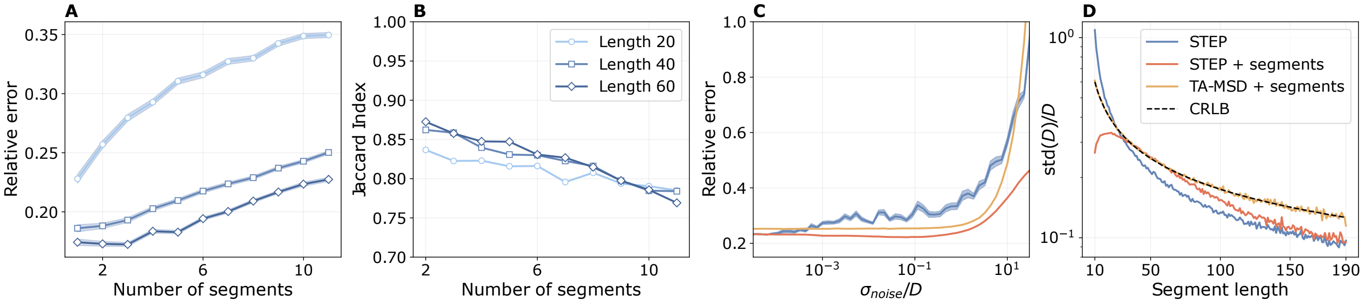

Figure 2. **STEP performance predicting the diffusion coefficient at every time step.** (**A**) Relative error of STEP as a function of the number of segments at three different segment lengths. (**B**) Prediction accuracy of STEP as a function of the number of segments at three different segment lengths. (**C**) Relative error of STEP (blue), STEP with known segments (red), and TA-MSD with known segments (yellow) as a function of the ratio between the localization noise's standard deviation σ and the diffusion coefficient D. (**D**) Prediction uncertainty as a function of the segment length for each of the previous methods compared to the Cramér-Rao lower bound (CRLB).

score for shorter segments when the trajectories are very long (11 segments). This suggests that every additional changepoint in the trajectory adds a similar amount of error sources which are eventually outweighed by the accumulated errors along the trajectory as it gets longer. Nonetheless, even in the most challenging cases with 11 segments, STEP correctly detect the vast majority of the points.

Thus, we study the resilience of our method to noise. In experimental scenarios, trajectories are affected by localization noise, which is usually modeled as Gaussian noise of variance σ^2_{noise} added to the trajectories. Since we consider diffusion coefficients at very different scales along the trajectories, in Fig. 2C, we plot the error as a function of the ratio between the noise's standard deviation and the diffusion coefficient. We see that STEP strongly outperforms the TA-MSD approach with known segments even well beyond the noise levels present in relevant experimental scenarios (usually $\sigma_{\text{noise}}/D < 10^{-1}$). Surprisingly, STEP can correctly extract the diffusion coefficient of constant segments (red line) even in the presence of large noise ($\sigma_{\text{noise}}/D > 10$).

Finally, we investigate STEP's ability to estimate D with higher precision that the TA-MSD approach (as shown in Fig. 2B), which is considered the optimal estimator for this calculation as documented by Michalet et al. [23]. To assess the prediction uncertainty of various methods, we examine it in relation to segment length in Fig. 2D. Our observations confirm that the TA-MSD fit is an optimal, unbiased estimator for D as it aligns closely with the Cramér-Rao lower bound (CRLB) (yellow and dashed black lines). Interestingly, STEP consistently deviates from the CRLB (blue and red lines below the dashed black line), suggesting a degree of bias in its estimation.

Several factors can contribute to introducing bias in the resulting model. For example, STEP estimates the logarithm of D, which can introduce bias since the use of logarithms typically leads to estimators with reduced variance. It is also essential to note that the model's accuracy heavily depends on both the training data and the loss function employed during training. In this specific case, the distribution of segment lengths in the training dataset follows an exponential function, resulting in a higher proportion of shorter segments. From the perspective of minimizing loss, there is a tendency to prioritize accuracy improvement for the shorter segments, even if it comes at the expense of accuracy for the longer ones.

Appendix D: Anomalous diffusion exponent prediction for various diffusion models

In the Results section, we briefly show how to use STEP to study particles that randomly switch between anomalous diffusing states. Here, we thoroughly characterize the suitability of the method for such task. We use STEP to predict the anomalous diffusion exponent α at every time step of trajectories composed of segments with constant anomalous diffusion exponent and diffusion model, as we detail in Appendix B 3.

In the main text, we have already studied how the mean absolute error (MAE) in the α prediction depends on the segment length, and we have compared STEP to two reference methods. As an additional reference, we obtain an MAE over all trajectories and time steps of 0.271 with STEP, 0.275 with STEP and known segments, 0.368 with TA-MSD and known segments, and CONDOR achieved an MAE of 0.237 for trajectories of the same lengths in the AnDi Challenge [3].

Furthermore, we can look at the performance segregated by the diffusion model. We report the MAE over all segments belonging to each anomalous diffusion model in Fig. 3A. We observe clear differences for some of the models with CTRW, FBM, and LW segments holding the lowest errors. Additionally, we provide a histogram of predicted and true α for all diffusion models in Fig. 4.

In particular, the MAE in scaled Brownian motion (SBM) segments is significantly larger than in the other

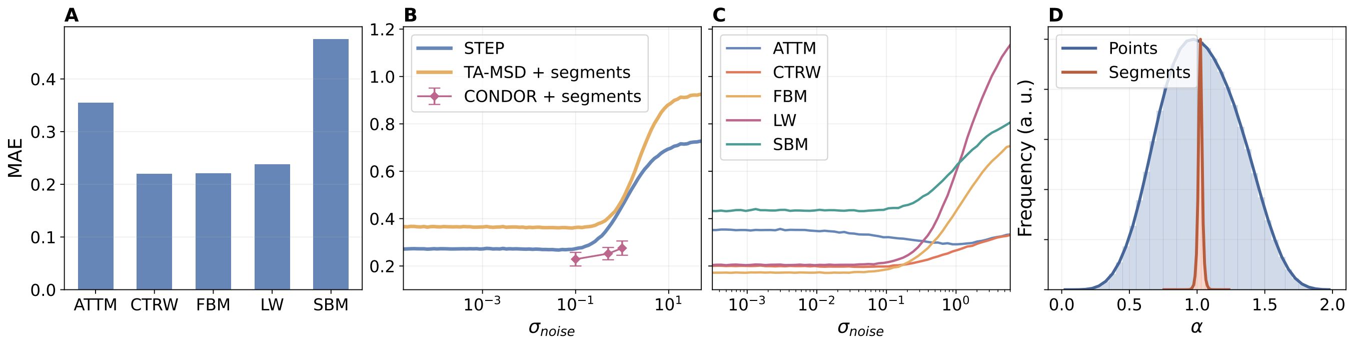

Figure 3. **STEP performance to predict α in terms of the mean absolute error (MAE).** We consider the anomalous diffusion models: ATTM, CTRW, FBM, LW and SBM (see Appendix B 3). (**A**) Prediction MAE by diffusion model. (**B**) MAE as a function of the localization noise for STEP, TA-MSD, and CONDOR, the latter two with known segments. (**C**) MAE as a function of the localization noise for STEP separated by the diffusion model. The blue line in (**B**) corresponds to the average of the lines presented in this panel. (**D**) Prediction of α for Brownian motion trajectories. The blue distribution shows the pointwise prediction for the trajectories, while the red one shows the mean prediction over trajectories. The distributions have been normalized to have the same maximum value.

models. This has already been observed in previous works (see for instance Fig. 2d of Ref. [3]), although the differences here are larger. A detailed inspection shows that the biggest errors come from shorter segments, in agreement with the results from Fig. 2D. This is reasonable since the aging in SBM is the source of the anomalous diffusion [1] and therefore it requires longer segments to be correctly characterized. It is also reasonable to expect the largest errors to happen whenever $\alpha \in (0, 2)$ is close to its range limits and the predictions are in the opposite side, which is mitigated in models with restricted ranges of α.

STEP displays a clear tendency to predict $\alpha \sim 0.8$ for SBM segments, as we can see in the right-most column of Fig. 4. This behavior is enhanced by the presence of noise, suggesting that the model struggles to identify any clear behavior in short segments, which also happen to be the most common.

Interestingly, we find a similar trend in CTRW segments, where the model has a tendency to predict $\alpha \simeq 0.25$, corresponding to nearly immobile particles. CTRW trajectories are characterized by jumps at random times, resulting in segments in which the particle does not move, usually referred to as waiting times. Hence, many CTRW segments in our heterogeneous trajectories do not display any movement due to their short lengths, corresponding to a waiting time section. Therefore, it is impossible for the model to correctly predict α, as it does not have any information to work with.

To a lesser extent, we also find that the model predicts $\alpha \sim 1$ for low anomalous diffusion exponents in ATTM segments. In ATTM trajectories with small α, we encounter very long segments with low diffusion coefficients. Similar to the CTRW case, we encounter parts of these long segments in our heterogeneous trajectories containing a unique diffusion coefficient, thus behaving like Brownian motion along the observed time window. Hence, the predictions $\alpha \sim 1$ are correct in these cases.

We proceed to study the resilience of the methods to localization noise, as we do in Appendix C. We present the MAE as a function of the noise standard deviation σ_{noise} in Fig. 3C. We observe a consistent performance of all the methods until reaching considerable levels of noise. Again, STEP is comparable to CONDOR despite the latter having the advantage of knowing the segments beforehand.

As we have seen throughout this section, characterizing some diffusion models is harder than others and the localization noise has a different impact on them, as we show in Fig. 3D and Fig. 4. While increasing the noise level has an overall negative effect, we see that the performance on LW segments suffers the most, while the performance on CTRW segments is barely affected. Overall, the errors start to increase significantly beyond $\sigma_{\text{noise}} \sim 2 \times 10^{-1}$, which would correspond to harsh experimental conditions. Interestingly, ATTM segments see a drop in MAE with increasing noise for a limited range.

In conclusion, we analyze the predictions of α for Brownian motion trajectories featuring random diffusion changes, as illustrated inFig. 2A & B. The pointwise prediction (blue distribution) averages to $\alpha = 1.02$, which aligns closely with the expected value of 1. This outcome gains further credibility when we compute the average predicted α across the entire trajectory (red distribution), since it shows that all trajectories are consistently predicted to have α values approximating 1. Although these trajectories closely resemble those of ATTM, characterized by random changes in diffusion coefficients, our model correctly discerns that the trajectories in question do not exhibit anomalous diffusion. This determination is based on the fact that the diffusion coefficients and dwell times do not satisfy the conditions outlined in Fig. 4 and related text, which are necessary to induce anomalous behavior.

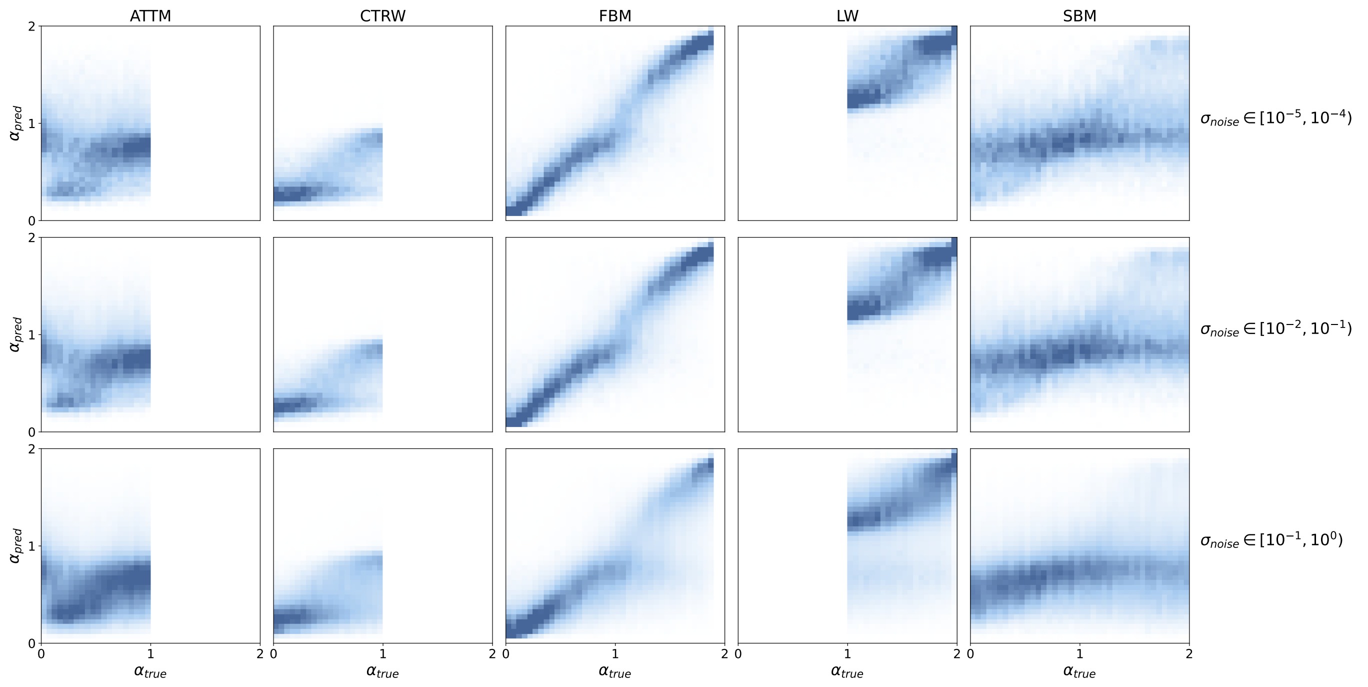

Figure 4. **Predicted vs true anomalous diffusion exponent.** 2D histograms showing the true and predicted anomalous diffusion exponents for the different diffusion models. Each column contains the histograms belonging to a different diffusion model for three different localization noise levels: $\sigma_{\text{noise}} \in [-5, -4)$ (top), $\sigma_{\text{noise}} \in [-2, -1)$ (middle), and $\sigma_{\text{noise}} \in [-1, 0)$ (bottom). The low-noise histograms (top row) result in Fig. 2C when combined together.

Appendix E: Experimental materials and methods

1. Cell culture and plating

For the live-cell single-molecule imaging experiments involving the integrin $\alpha5\beta1$, HeLa cells were cultured in DMEM (Gibco, 11960-044), supplemented with 10% (v/v) fetal bovine serum (FBS, Sigma). Cells were tested for mycoplasma contamination using PCR (Biotools kit, 4542). For fluorescence imaging, glass-bottom dishes (IBIDI, 81158) were coated with fibronectin (FN, Sigma, F2008) by placing 10 μg/mL FN on the glass for 1 h at 37 °C, and then blocked with BSA 2 mg/mL for 1 h at 37 °C. Cells were plated at a density of 5×10^4 cell/dish and cultured for 24 h prior to use.

2. Preparation of half-antibody fragments

Half-antibody fragments were obtained following a protocol similar to the one used in [24]. Briefly, mouse anti-human integrin $\alpha5$ antibody (50 μL; BD Biosciences, 610633) was dialyzed (ThermoFisher, Slide-A-Lyze MINI Dialysis Device, 2K) against PBS overnight at room temperature to replace the commercial buffer. Then, antibodies were reduced with 1 mM DTT for 30 min at room temperature and dialyzed again, using Slide-A-Lyze MINI Dialysis Device 2K, for 4 h at room temperature against PBS to remove DTT. To avoid reassociation of reduced antibodies, sulfhydryl groups were blocked by incubating with iodoacetamide 20 mM for 1 h at 4 °C with agitation. Iodoacetamide was then removed from the reaction by dialysis overnight at 4 °C. Finally, reduced antibodies were biotinylated with a 10-fold molar excess of EZ-Link Sulfo-NHS_LC_Biotin (Thermo Scientific) for 30 min at room temperature with agitation and stored at 4 °C until use.

3. Single-molecule labeling

Biotinylated half-antibody fragments were conjugated to streptavidin-coated quantum dots (QD655 streptavidin conjugate, Invitrogen, Q10123mp). Cells were washed 3 times with washing buffer (PBS with 6% BSA) and labeled with half antibody-quantum dots (about 1 nM) in washing buffer (200 μL per dish) for 15 min at 37 °C, followed by two washes.

4. Live-cell single-molecule imaging

Imaging was performed using a Leica DMi8 fluorescence microscope. Samples were illuminated in total internal reflection fluorescence (TIRF) geometry. Excitation was achieved with a CW laser (Obis, Coherent, λ=488 nm, <1 kW/cm^2). Fluorescence was recorded using an oil-immersion objective (Leica, 100X,

NA=1.47) and an sCMOS camera (Photometrics 95B) with appropriate filters (Chroma). Movies were recorded at a frame rate of 33 Hz. A microscope environment chamber (Okolab) was used to keep cells in a 5% CO_2 atmosphere while recording.

5. Single-particle tracking

Particle detection and tracking were performed using u-track [25]. The detection (Gaussian Mixture-Model Fitting) and tracking parameters were optimized based on visual inspection and performance diagnostic of the resulting detection and tracking. All image and data analysis tasks were performed in MATLAB 2020a and more recent versions (The MathWorks, Natick, MA). Videos were loaded into MATLAB using Bio-Formats [26].

[1] Ralf Metzler, Jae-Hyung Jeon, Andrey G Cherstvy, and Eli Barkai. Anomalous diffusion models and their properties: non-stationarity, non-ergodicity, and ageing at the centenary of single particle tracking. *Physical Chemistry Chemical Physics*, 16(44):24128–24164, 2014.

[2] Joseph Klafter and Igor M Sokolov. *First steps in random walks: from tools to applications.* OUP Oxford, 2011.

[3] Gorka Muñoz-Gil, Giovanni Volpe, Miguel Angel Garcia-March, Erez Aghion, Aykut Argun, Chang Beom Hong, Tom Bland, Stefano Bo, J Alberto Conejero, Nicolás Firbas, et al. Objective comparison of methods to decode anomalous diffusion. *Nature Communications*, 12:6253, 2021.

[4] Pietro Massignan, Carlo Manzo, Juan A Torreno-Pina, Maria F García-Parajo, Maciej Lewenstein, and GJ Lapeyre Jr. Nonergodic subdiffusion from brownian motion in an inhomogeneous medium. *Physical Review Letters*, 112(15):150603, 2014.

[5] Harvey Scher and Elliott W. Montroll. Anomalous transit-time dispersion in amorphous solids. *Phys. Rev. B*, 12:2455–2477, Sep 1975.

[6] Benoit B. Mandelbrot and John W. Van Ness. Fractional brownian motions, fractional noises and applications. *SIAM Review*, 10(4):422–437, 1968.

[7] J. Klafter and G. Zumofen. Lévy statistics in a hamiltonian system. *Phys. Rev. E*, 49:4873–4877, Jun 1994.

[8] S. C. Lim and S. V. Muniandy. Self-similar gaussian processes for modeling anomalous diffusion. *Phys. Rev. E*, 66:021114, Aug 2002.

[9] Borja Requena and Gorka Muñoz-Gil. Step python library (https://github.com/borjarequena/step), December 2022.

[10] Adam Paszke, Sam Gross, Francisco Massa, Adam Lerer, James Bradbury, Gregory Chanan, Trevor Killeen, Zeming Lin, Natalia Gimelshein, Luca Antiga, Alban Desmaison, Andreas Kopf, Edward Yang, Zachary DeVito, Martin Raison, Alykhan Tejani, Sasank Chilamkurthy, Benoit Steiner, Lu Fang, Junjie Bai, and Soumith Chintala. Pytorch: An imperative style, high-performance deep learning library. In *Advances in Neural Information Processing Systems*, volume 32. Curran Associates, Inc., 2019.

[11] Jeremy Howard and Sylvain Gugger. Fastai: A layered api for deep learning. *Information*, 11(2), 2020.

[12] Charles Truong, Laurent Oudre, and Nicolas Vayatis. Selective review of offline change point detection methods. *Signal Processing*, 167:107299, 2020.

[13] Tong He, Zhi Zhang, Hang Zhang, Zhongyue Zhang, Junyuan Xie, and Mu Li. Bag of tricks for image classification with convolutional neural networks. In *Proceedings of the IEEE/CVF Conference on Computer Vision and Pattern Recognition*, pages 558–567, 2019.

[14] Ashish Vaswani, Noam Shazeer, Niki Parmar, Jakob Uszkoreit, Llion Jones, Aidan N Gomez, Łukasz Kaiser, and Illia Polosukhin. Attention is all you need. In *Advances in Neural Information Processing Systems*, volume 30, 2017.

[15] Diederik P. Kingma and Jimmy Ba. Adam: A method for stochastic optimization. In *ICLR (Poster)*, 2015.

[16] Leslie N. Smith. A disciplined approach to neural network hyper-parameters: Part 1 – learning rate, batch size, momentum, and weight decay. *arXiv preprint arXiv:1803.09820*, 2018.

[17] Leslie N. Smith and Nicholay Topin. Super-convergence: very fast training of neural networks using large learning rates. In *Artificial Intelligence and Machine Learning for Multi-Domain Operations Applications*, volume 11006, pages 369 – 386. International Society for Optics and Photonics, SPIE, 2019.

[18] Geoffrey E. Hinton, Nitish Srivastava, Alex Krizhevsky, Ilya Sutskever, and Ruslan R. Salakhutdinov. Improving neural networks by preventing co-adaptation of feature detectors. *arXiv preprint arXiv:1207.0580*, 2012.

[19] Stephen Hanson and Lorien Pratt. Comparing biases for minimal network construction with back-propagation. In *Advances in Neural Information Processing Systems*, volume 1, 1988.

[20] Anders Krogh and John Hertz. A simple weight decay can improve generalization. In *Advances in Neural Information Processing Systems*, volume 4, 1991.

[21] Carlo Manzo, Juan A Torreno-Pina, Pietro Massignan, Gerald J Lapeyre Jr, Maciej Lewenstein, and Maria F Garcia Parajo. Weak ergodicity breaking of receptor motion in living cells stemming from random diffusivity. *Physical Review X*, 5(1):011021, 2015.

[22] Gorka Muñoz-Gil, Borja Requena, Giovanni Volpe, Miguel Angel Garcia-March, and Carlo Manzo. Andi_datasets python library (github.com/andichallenge/andi_datasets), 2021.

[23] Xavier Michalet and Andrew J Berglund. Optimal diffusion coefficient estimation in single-particle tracking. *Physical Review E*, 85(6):061916, 2012.

[24] Shalini T Low-Nam, Keith A Lidke, Patrick J Cutler, Rob C Roovers, Paul MP van Bergen en Henegouwen, Bridget S Wilson, and Diane S Lidke. Erbb1 dimerization is promoted by domain co-confinement and stabilized by ligand binding. *Nature Structural & Molecular Biology*, 18(11):1244–1249, 2011.

[25] Khuloud Jaqaman, Dinah Loerke, Marcel Mettlen, Hirotaka Kuwata, Sergio Grinstein, Sandra L Schmid, and Gaudenz Danuser. Robust single-particle tracking in live-cell time-lapse sequences. *Nature Methods*, 5(8):695–702, 2008.

[26] Melissa Linkert, Curtis T Rueden, Chris Allan, Jean-Marie Burel, Will Moore, Andrew Patterson, Brian Loranger, Josh Moore, Carlos Neves, Donald MacDonald, et al. Metadata matters: access to image data in the real world. *Journal of Cell Biology*, 189(5):777–782, 2010.
